# Genetic dissection of dopaminergic and noradrenergic contributions to catecholaminergic tracts in early larval zebrafish

**DOI:** 10.1002/cne.22214

**Published:** 2009-08-16

**Authors:** Edda Kastenhuber, Claudius F Kratochwil, Soojin Ryu, Jörn Schweitzer, Wolfgang Driever

**Affiliations:** 1 Developmental Biology, Institute of Biology I, University of FreiburgD-79104 Freiburg, Germany; 2 Freiburg Institute for Advanced Studies, University of FreiburgD-79104 Freiburg, Germany

**Keywords:** dopaminergic neurons, noradrenergic neurons, catecholaminergic projections, ascending projections, diencephalospinal projections

## Abstract

The catecholamines dopamine and noradrenaline provide some of the major neuromodulatory systems with far-ranging projections in the brain and spinal cord of vertebrates. However, development of these complex systems is only partially understood. Zebrafish provide an excellent model for genetic analysis of neuronal specification and axonal projections in vertebrates. Here, we analyze the ontogeny of the catecholaminergic projections in zebrafish embryos and larvae up to the fifth day of development and establish the basic scaffold of catecholaminergic connectivity. The earliest dopaminergic diencephalospinal projections do not navigate along the zebrafish primary neuron axonal scaffold but establish their own tracts at defined ventrolateral positions. By using genetic tools, we study quantitative and qualitative contributions of noradrenergic and defined dopaminergic groups to the catecholaminergic scaffold. Suppression of Tfap2a activity allows us to eliminate noradrenergic contributions, and depletion of Otp activity deletes mammalian A11-like Otp-dependent ventral diencephalic dopaminergic groups. This analysis reveals a predominant contribution of Otp-dependent dopaminergic neurons to diencephalospinal as well as hypothalamic catecholaminergic tracts. In contrast, noradrenergic projections make only a minor contribution to hindbrain and spinal catecholaminergic tracts. Furthermore, we can demonstrate that, in zebrafish larvae, ascending catecholaminergic projections to the telencephalon are generated exclusively by Otp-dependent diencephalic dopaminergic neurons as well as by hindbrain noradrenergic groups. Our data reveal the Otp-dependent A11-type dopaminergic neurons as the by far most prominent dopaminergic system in larval zebrafish. These findings are consistent with a hypothesis that Otp-dependent dopaminergic neurons establish the major modulatory system for somatomotor and somatosensory circuits in larval fish. J. Comp. Neurol. 518:439–458, 2010. © 2009 Wiley-Liss, Inc.

The major catecholamines (CA) used as neurotransmitters by the central nervous system (CNS) are dopamine and noradrenaline (Smeets and González,^2000^; Björklund and Dunnett, 2007). The dopaminergic (DA) systems have broad functions in the brain, including motor activity, perception, and sleep, and, at least in mammals, they are involved in motivational behavior, mood, reward, learning, and attention (Schultz,^2007^; Sulzer,^2007^). Noradrenergic (NA) neurons play an important role in the control of autonomic systems, such as heart rate, blood pressure, and glucose release. It regulates diverse processes in the brain, such as attention or arousal, and influences the reward system (Ordway et al.,^2007^), as with dopamine. Both neurotransmitters have received much attention in the last decades, particularly because of their early-recognized involvement in psychiatric and neurological disorders, such as Parkinson's disease, schizophrenia, bipolar disorder, and restless legs syndrome (Hirsch et al.,^1988^; Lang and Lozano,^1998^; Clemens et al.,^2006^). This wide spectrum of activities correlates with the observation that CA cell groups send extensive and far-ranging projections within the brain and to the spinal cord. Although the anatomy of CA neurons and axonal tracts has been extensively studied in amniote vertebrates (Smeets and González,^2000^), their development is less well understood. For genetically and experimentally tractable vertebrate model organisms, such as the zebrafish, many aspects of CA axonal tract formation have to be investigated before a detailed analysis of developmental mechanisms and correlation with circuit function can be achieved.

The mammalian CA cell groups in the CNS are numbered in a caudal-to-rostral order, as introduced by Dahlstroem and Fuxe (1964) and refined later by Hökfelt et al. (1984). The mammalian noradrenergic groups A1, A2, and A4–A7 are located in the hindbrain. The caudal noradrenergic groups, A1 and A2, of the medulla oblongata (MO) have ascending projections to the forebrain and primarily to the hypothalamus (Smeets and González,^2000^). The most prominent noradrenergic neurons reside more rostrally in the locus coeruleus (LC, A6) and have both ascending and descending projections. Thus, noradrenergic projections innervate most parts of the CNS, among others the spinal cord, cerebellum, hypothalamus, thalamus, hippocampus, striatum, and neocortex (Ordway et al.,^2007^). In contrast, the fibers of the A5 and A7 cell groups do not build such a wide network as the A6 group of the LC. They project mainly inside the brainstem and to spinal cord regions (Smeets and González,^2000^).

The dopaminergic cells of the retrorubal area (A8), substantia nigra pars compacta (A9), and ventral tegmental area (A10) represent the major dopaminergic groups of mammals. In contrast to the wide distribution of NA axons, mesencephalic DA neurons send out topographically well-delineated, ascending projections to forebrain targets via the mesocortical, mesolimbic, and mesostriatal (or nigrostriatal) pathways (for review see Björklund and Dunnett, 2007), which are considered to be three of the four major far-reaching dopaminergic pathways exerting the effects of dopamine. Dopaminergic neurons are also located in the diencephalon (A11–A15), olfactory bulb (A16), and inner nuclear layer of the retina (A17). A12 cell group DA neurons projecting to the median eminence and to the hypophysis constitute the tuberoinfundibular system, the fourth major dopaminergic pathway that controls the release of prolactin. The diencephalospinal DA projections emanate primarily from the A11 DA cell group (Björklund and Skagerberg,^1979^; Gunnar and Olle,^1985^). The incertohypothalamic CA system consists mainly of projections from A13 DA neurons, which are joined by axons of DA groups A11 and A14 to target regions in the hypothalamus. Finally, projections of the A14 DA cell group form the periventricular dopaminergic system and innervate, apart from the periventricular and preoptic area, the hypophysis as well. The DA cells in the olfactory bulb and retina build only local circuits (Smeets and González,^2000^).

The zebrafish (*Danio rerio*) is an excellent model organism for studying vertebrate brain development. A functional nervous system is established after 4–5 days of larval development and allows zebrafish larvae to perform complex behaviors such as swimming and hunting (Kuwada,^1995^; Budick and O'Malley,^2000^). The large number, fast external development, and easy accessibility of the transparent embryos, together with the established genetics, are the main advantages of studying CA system development in zebrafish. However, teleost and thus zebrafish brain architecture and circuit formation are different from those of mammals. Most significantly, the zebrafish lacks dopaminergic neurons in the mesencephalon (Meek,^1994^; Smeets and Reiner,^1994^; Holzschuh et al.,^2001^; Kaslin and Panula,^2001^). Similarly to the case in amniote vertebrates, DA cell groups have been described in the olfactory bulb, subpallium (ventral telencephalon), retina, preoptic region, pretectum, and ventral diencephalon, including the hypothalamus (Holzschuh et al.,^2001^; Kaslin and Panula,^2001^; Rink and Wullimann,^2001^; Ma and Lopez,^2003^; McLean and Fetcho,2004a). At larval stages, the most prominent DA neurons form in the ventral diencephalon and have been subdivided further according to the shape and location of cell groups in the ventral thalamus (group 1), posterior tuberculum (groups 2 and 4), and medial (group 3) and lateral (groups 5 and 6) hypothalamus (Rink and Wullimann,2002a). For some zebrafish DA groups, the probability with which they are homologous to the mammalian dopaminergic subgroups remains elusive. A comparative analysis of the evolution of dopaminergic systems from teleosts to mammals suggests that mesencephalic dopaminergic neurons arose in the tetrapod lineage subsequent to caudal expansion or migration of ventral diencephalic DA cell groups, implying a close evolutionary relationship between these DA cell groups (Smeets and González,^2000^). However, there are also other explanations for the absence of midbrain DA neurons in teleost fish. Cartilaginous fish possess basal DA midbrain neurons (Stuesse et al.,^1994^). Therefore, it is possible that they arose within gnathostomes and later were lost in teleosts for as yet unknown reasons. Another explanation would be independent evolution of midbrain DA neurons in cartilaginous fish and tetrapods. At least some developmental control mechanisms of the mammalian A11 group providing the DA diencephalospinal tract seems to be conserved between zebrafish and mouse (Ryu et al.,^2007^). NA cell groups are located in the LC, MO, and area postrema (AP; Ma,1994a,b,^1997^), as in mammals.

CA axonal projections have been described for adult (Ma,1994b,^1997^; Kaslin and Panula,^2001^; Rink and Wullimann,^2001^,2002a,b; Ma,^2003^) and larval (Rink and Wullimann,2002b; McLean and Fetcho,2004a; Sallinen et al.,^2009^) zebrafish. In these studies, antibodies against tyrosine hydroxylase (TH) labeling both DA and NA neurons and antibodies against dopamine beta hydroxylase (DBH) specific for NA cells have been used on zebrafish brain sections to differentiate between NA and DA systems. However, it has been impossible so far to visualize the NA projection positively; although the DBH antibody marks cell bodies of NA neurons, their axonal projections are only poorly labeled. A dissection of dopaminergic and noradrenergic contributions to the distinct CA circuits could clarify the proportion and connectivity of specific CA neuron populations. We therefore decided to take a genetic approach to distinguish DA and NA projections. We utilized genetic mutations and morpholino knockdown of *orthopedia* (*otp*) genes to eliminate DA neurons with descending and ascending projections and of *tfap2α* to eliminate NA neuronal projections of the hindbrain. This enabled us to visualize the relative contributions of NA and DA systems to ascending and descending CA projections. We were able to demonstrate the NA and DA contributions to the anterior CA tract and the medial longitudinal CA tract. Furthermore, our results reveal that DA neurons of the posterior tuberculum provide the majority of descending and ascending CA projections in larval zebrafish.

## MATERIALS AND METHODS

### Fish maintenance and genetic lines

Zebrafish breeding and maintenance were carried out under standard conditions at 28.5°C (Westerfield,^1994^). To inhibit pigmentation, we incubated embryos in 0.2 mM 1-phenyl-2 thiourea. Experiments were performed with AB/TL wild-type fish and *tfap2α*^*m819*^ (Holzschuh et al.,2003a) and *otpa*^*m866*^ (Ryu et al.,^2007^) mutant strains. All the experimental procedures were in accordance with the German laws for animal care.

### Genotyping

The *otpa*^*m866*^ mutant fish were genotyped by genomic PCR with a common reverse primer and mismatch forward primers whose 3′-end preferentially hybridizes with mutant or wild-type sequences (wild-type forward primer, 5′-GTAGCGGTCAACAGTAAGGATCAATA-3′; mutant forward primer, 5′-GTAGCGGTCAACAGTAAGGATCAACG-3′; and common reverse primer, 5′-CGTTAAGCTGAGCCGGAGTAAAGC-3′; Ryu et al.,^2007^). Fish carrying the *tfap2α*^*m819*^ mutation were identified by genomic PCR using primers flanking exon 5 (forward, 5′-TTATTATGCTCACGCGCTCA-3′; reverse, 5′-TTGCAAAACAGACACTCTCCA-3′) and subsequent DraII restriction digest of the amplified DNA. Amplicons of *tfap2α*^*m819*^ allele but not wild-type *tfap2α* allele were cleaved into two fragments of 123 bp and 237 bp (Holzschuh et al.,2003a).

### Markers

Antibody markers used in this study were as follows.

#### Anti-TH antibody

**Figure 1:**
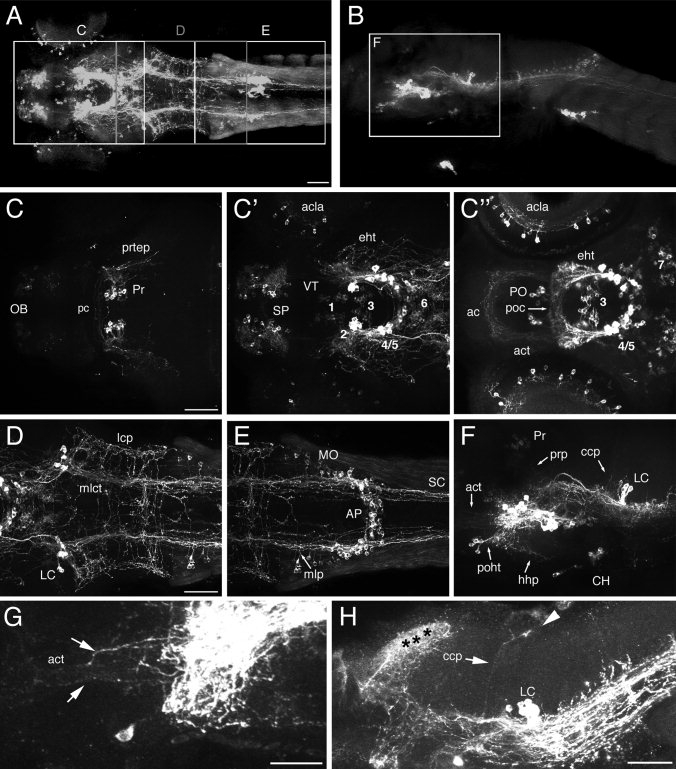
CA projections in 3-day-old wild-type zebrafish early larvae. Z-projections of confocal stacks of whole-mount anti-TH immunohistochemistry in 72 hpf (A–G) or 5 dpf (H) wild-type embryos are shown. Dorsal (A,C–E) and lateral (B,F–H) views, anterior to the left (A–H), dorsal at top (B,F–H). **A:** Z-projection (189 μm) showing a dorsal overview of TH immunoreactivity. Regions depicted in C–E are indicated by boxes. The faint signal in somites is nonspecific. **B:** Z-projection (105 μm) showing a lateral overview of TH immunoreactivity. The box illustrates the region depicted in F. **C–C′′:** Z-projections (65 μm, 90 μm, and 50 μm, respectively) of anterior brain regions going from dorsal (C) to ventral (C′′) showing THir circuitry in the tel- and diencephalon. C: Projections of the pretectal THir cluster are indicated. The olfactory bulb THir neurons project only locally. C′: THir tracts of the retina, ventral diencephalon, and subpallium. C′′: Ventral diencephalon including preoptic region and hypothalamus. Commissures and tracts containing THir fibers are labeled. **D:** Z-projection (125 μm) showing the most posterior groups in the diencephalon and the LC in the rhombencephalon. **E:** Z-projection (121 μm) of posterior rhombencephalon and spinal cord. **F:** Z-projection (105 μm) of lateral confocal stack of the larval brain. **G:** Z-projection of a lateral confocal stack showing the act (arrows point to axons belonging to the act). **H:** Z-projection of a lateral confocal stack demonstrating the ccp (arrow) projecting to the cerebellum (arrowhead). Asterisks indicate dense innervation of THir fibers in the tectum. The numbering of ventral diencephalic DA groups is according to Rink and Wullimann (2002a). For abbreviations see list. Scale bars = 50 μm in A (applies to A,B); 50 μm in C; 50 μm in D (applies to D–F); 10 μm in G; 20 μm in H.

For labeling of CA neurons and their axonal projections, an anti-TH antibody was generated: a 824-bp fragment corresponding to nucleotides 274–1097 of the zebrafish *th1* gene (ensemble transcript NM_131149) was PCR amplified and cloned into the pTRC-hisB vector (Invitrogen, LaJolla, CA). Proteins were overexpressed in *Escherichia coli* by standard procedures and purified with an Ni^+^NTA column (Qiagen Germany) under denaturing conditions. Rabbit antiserum production was performed by Sigma Genosys (Cambridge, United Kinghdom). Terminal bleed of the immunized rabbit (No. 2914) was used in all experiments. The specificity of the serum was initially tested with a Western blot. The serum, at 1:200 dilution, recognized 2 ng of the recombinant TH1 protein as a specific band on the Western blot. For whole-mount immunohistochemistry, we further processed the antiserum by extensive preabsorption against fixed 18–24-hours-postfertilization (hpf) zebrafish embryos, stages at which no or very little TH1 is expressed (50 μl serum preabsorbed against about 500 embryos in blocking solution). To prove further the specificity of the antibody, we performed fluorescent in situ hybridization to *th1* combined with TH1 immunohistochemistry, which revealed specific labeling of DA/NA neurons (Supp. Info. Fig. 1), whereas omission of the primary antibody did not stain any cell somata that did not express *th1*.

#### Antiacetylated α-tubulin antibody

For labeling of the early zebrafish axonal scaffold, we used a mouse monoclonal antiacetylated α-tubulin antibody (1:500; clone 6-11B-1; reference T 6793; Sigma, St. Louis, MO). This antibody was raised against acetylated α-tubulin of a sea urchin and has been used for detection of acetylated tubulin from many organisms, including zebrafish. The staining achieved with this antibody was a cytoplasmic staining similar to that obtained in previous studies in zebrafish (Chitnis and Kuwada,^1990^).

#### Anti-zn-5 (DM-GRASP)

For labeling of retinal ganglion cells, secondary motorneurons, cranial ganglia, and their axons we used a monoclonal anti-DM-GRASP antibody (1: 4,000, zn-5 antibody; Zebrafish International Resource Center). This antibody was raised against adult zebrafish hindbrain membranes (Trevarrow et al.,^1990^). Peptide sequencing of purified zn-5 antigen revealed that the zn-5 antibody binds specifically to DM-GRASP (Fashena and Westerfield,^1999^).

The following secondary antibodies were used: AlexaFluor 555 F(ab)_2_ fragment of goat anti-rabbit IgG (H + L) at 1:1,000; this goat antiserum (Invitrogen, Paisley, Scottland; No. A-21430) was prepared against rabbit immunoglobulin G (heavy and light chain); AlexaFluor 488 F(ab)_2_ fragment of goat anti-mouse IgG (H + L) at 1:1,000; this goat antiserum (Invitrogen; No. A-11070) was prepared against mouse immunoglobulin G (heavy and light chain).

### Whole-mount immunohistochemistry

Embryos were fixed at desired time points in fresh buffered paraformaldehyde fixative (4% paraformaldehyde, 0.15 mM CaCl_2_, 4% sucrose, 1% dimethyl sulfoxide in 0.1 M PO_4_ buffer) for 2 hours at room temperature or alternatively for 4 hours at 4°C. Immunohistochemistry was adapted from a previous protocol (Schulter-Merker,^2002^), with the exception that fixed embryos were permeabilized by incubation with proteinase K (10 μmg/ml; Sigma). Proteinase K was successively inhibited by postfixation in 4% paraformaldehyde. We used rabbit polyclonal anti-TH primary antibody at 1:500 dilution (Ryu et al.,^2007^), which was detected with anti-rabbit Alexa555-conjugated secondary antibody (1:1,000; Invitrogen) TH is the rate-limiting enzyme in catecholamine biosynthesis and is expressed in DA and NA neurons. Double immunohistochemistry was performed with monoclonal mouse antiacetylated tubulin primary antibody at 1:500 dilution (Sigma) or mouse zn-5 primary antibody (against DM-GRASP) at 1:4,000 (Trevarrow et al.,^1990^) mixed with anti-TH primary antibody. The mouse antibodies were detected by anti-mouse Alexa488-conjugated secondary antibody (1:1,000; Invitrogen). Where indicated, embryos were counterstained with TOTO-3 (Invitrogen) to visualize all nuclei.

### Morpholino injections

The potency and specificity of *otpb* morpholino targeting the second exon–intron boundary (5′-GAGCAAGTTCATTAAGTCTCACCTG-3′; Ryu et al.,^2007^) and *tfap2α* morpholino (5′-CCTCCATTCTTAGATTTGGCCCTAT-3′) against the intron 5 splice acceptor junction (Knight et al.,^2003^) have been described previously (synthesized by Gene Tools, Philomath, OR). Approximately 1 nl of morpholino solution at a concentration of 1–4 ng/nl was injected into single embryos at the one-cell stage.

### Imaging

Confocal z-stacks were recorded using a Zeiss LSM 5 Duo laser scanning confocal microscope. Z-projections were made with LSM 510 software. Levels and contrast of pictures were adjusted and figures were composed in Adobe Photoshop 7.0 and CS2 software.

## RESULTS

### CA cell groups and projection tracts in wild-type zebrafish embryos

**Figure 2:**
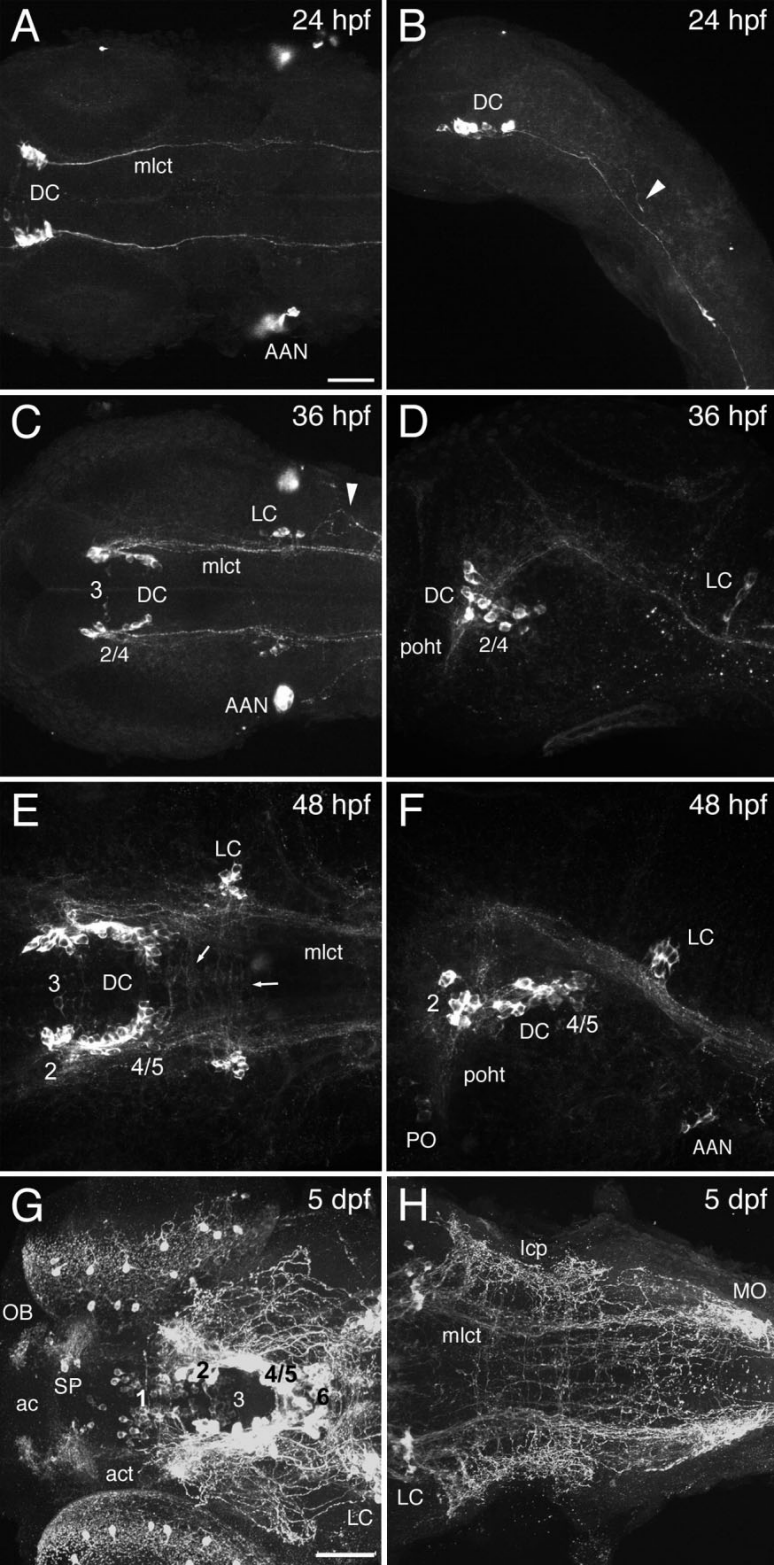
Formation of CA projections during development. Z-projections of confocal stacks of whole-mount anti-TH immunohistochemistry in wild-type embryos are shown; dorsal (A,C,E,G) and lateral (B,D,F,H) views, anterior to the left. **A:** Dorsal z-projection of a 24 hpf embryo shows that mlct axons are the first THir projections. **B:** Lateral z-projection of an embryo at 24 hpf. The arrowhead indicates a growth cone of an outgrowing mlct axon. **C,D:** Z-projections of TH immunoreactivity in 36 hpf embryos. Noradrenergic LC neurons are forming, and lcp axons start to arborize in the lateral hindbrain (arrowhead). **E:** Dorsal z-projection of a 48-hpf embryo. The first transversal THir projections appear in the region between the mid–hindbrain border and the LC. **F:** Lateral z-projection of an embryo at 48 hpf. The poht between the DC and the preoptic region is detectable. **G,H:** Z-projections of 5-dpf free-swimming larvae. The AAN expresses *th* already as migratory precursors and later contributes to the carotid body, which detects changes in blood flow composition. The numbering of ventral diencephalic DA groups is according to Rink and Wullimann (2002a). For abbreviations see list. Scale bars = 50 μm in A (applies to A–F); 50 μm in G (applies to G,H).

Visualization of CA tracts in zebrafish whole embryos and larvae allows identification of the projection patterns of the distinct CA neuronal groups. To this end, we have recently generated a polyclonal antityrosine hydroxylase antibody that made it possible to visualize even single axons by confocal microscopy (Ryu et al.,^2007^). Here, we use zebrafish mutations and morpholino-based knockdown of gene function to evaluate the contribution of DA and NA groups to CA TH-immunoreactive (THir) projections. Because morpholino knockdown is not reliable after 3 days of development, as a result of dilution and loss of morpholino, we focused our analysis on early larval stages at 72 hpf (Fig. 1). At this developmental stage, larvae have hatched, and most CA neuronal groups have formed (Holzschuh et al.,^2001^). Previous analysis of larval CA systems in zebrafish has focused on 5-days-postfertilization (dpf) larvae (McLean and Fetcho,2004a). Although more neurons are added to CA groups between 3 and 5 dpf, essentially all CA groups except for some local projecting hypothalamic groups already exist at 3 dpf, and extensive axonal projections have formed (Figs. 1, 2). With our focus on 3-dpf early larvae, we might also miss some late-forming CA neurons in the hindbrain, including small numbers of CA neurons that have been suggested to be dopaminergic in the area postrema (McLean and Fetcho,2004a). The state of the zebrafish CA systems at 3 dpf, as judged from anti-TH immunohistochemistry (TH-IHC) of whole larvae and confocal microscopy analysis, is presented in Figure 1, and the CA projections that can be identified at this stage are listed in Table 1. Figure 9 shows a schematic drawing of CA groups and projections.

**TABLE 1:** Nomenclature of Catecholaminergic Projections^1^

Description of tracts with CA contributions	Anatomical term	Abbreviation
Commissure in ventral telencephalon	Anterior commissure	ac
Commissure with contribution by preoptic DA neurons	Postoptic comissure	poc
Commissure in dorsal diencephalon	Posterior comissure	pc
Projections to the cerebellum	Cerebellar catecholaminergic projections	ccp
Projections from pretectum into tectum	Pretectotectal projections	prtep
Projections between ventral diencephalon and dorsal pretectum	Pretectal projections	prp
Projections between hypothalamus and hypophysis	Hypothalamic–hypophyseal projections	hhp
Lateral catecholaminergic projections in the hindbrain	Lateral CA projections	lcp
Local projections of the noradrenergic medulla neurons	Medulla local projections	mlp
Axonal tract between ventral diencephalon and ventral telencephalon	Anterior catecholaminergic tract	act
Tract between preoptic region and ventral diencephalon	Preopticohypothalamic tract	poht
Tract interconnecting groups in posterior tuberculum and hypothalamus	Endohypothalamic tract	eht
Medial longitudinal catecholaminergic tract to hindbrain and spinal cord	Medial longitudinal CA tract	mlct
Local arbors of the olfactory bulb	Local arbors of olfactory bulb	obla
Local arbors of amacrine cells in the retina	Local arbors of amacrine cells	acla

1 Catecholaminergic projections are listed with their anatomical description, anatomical term, and the abbreviation used. This list refers to anatomical terms used by Ma (1994b,^1997^,^2003^), Rink and Wullimann (2001,2002a), McLean and Fetcho (2004a), and Chitnis and Kuwada (1990).

Based on the genome duplication that occurred during early teleost evolution, two paralogous *th* genes, termed *th1* and *th2*, have recently been reported for zebrafish (Candy and Collet,^2005^). Because of protein sequence divergence, the previously used antibodies, which were generated against *th1* (Ryu et al.,^2007^) or mammalian *th*, might not have recognized the second paralog. The accompanying paper (Filippi et al., this issue), reports expression of *th2* in larval and juvenile zebrafish and reveals that the anti-TH1 antibody does not recognize TH2 protein. However, during embryonic and early larval stages, there is only a very small number of cells expressing *th2*, which are located predominantly in the caudal hypothalamus. Therefore, *th2*-expressing neurons have not been investigated here.

In the telencephalon, DA groups are present in the olfactory bulb (OB) and subpallium (SP). Olfactory DA neurons send out short arborizing axons (olfactory bulb local arbors, obla; Fig. 1C). Analyzing the projections of the subpallial DA neurons, we observed locally projecting fibers as well as ventrolateral projecting axons (Fig. 1C′) The retina also contains THir cells in the inner nuclear layer, which arborize locally (amacrine cells local arbors, acla; Fig. 1C′,C′′). The dorsalmost diencephalic DA neurons form in the pretectum and appear to project reciprocally to each other by THir axons through the posterior commissure (pc). In addition, the pretectal DA neurons send projections to the tectum (pretecto–tectal projections, prtep; Fig. 1C) and are also linked to the ventral diencephalon (DC) by THir pretectal projections (prp; Figs. 1F, 5C).

Axon tracts extending between the prominent DA cell clusters of the posterior tuberculum and hypothalamus establish the endohypothalamic tract (eht; Fig. 1C′). Two commissures containing THir axons, the anterior (ac) and the postoptic commissure (poc), cross the midline in the ventral tel- and diencephalon, respectively (Fig. 1C′′). In the proximity, a longitudinal THir tract between the ventral diencephalon and the subpallium has been named the anterior CA tract (act; Fig. 1C′′,G). The preopticohypothalamic tract (poht) is the projections between DA cells in the preoptic region and the ventral diencephalon (Fig. 1F). THir axons extending in the direction of the hypophysis were called hypothalamic–hypophyseal CA projections (hhp; Fig. 1F).

The predominant THir axons are concentrated in the medial longitudinal CA tract (mlct), which originates in the diencephalon, passes the noradrenergic LC neurons, and projects through the hindbrain into the spinal cord (Fig. 1D,E). At the level of the LC, an ascending THir projection toward the cerebellum (cerebellar CA projection, ccp) can be detected (Fig. 1F,H). The lateral CA projections (lcp) comprise THir projections lateral to the mlct in a region between the LC and the MO (Fig. 1D). THir axons connecting the right and left mlct characteristically cross the midline between caudal diencephalon and MO. Such transversal THir fibers were never found in the spinal cord (Fig. 1D,E). NA axons from the LC contribute to the mlct, whereas noradrenergic MO neurons extend local MO local projections (mlp) projections and may also contribute to mlct (Fig. 1D–F).

### Ontogeny of CA systems in zebrafish embryos

To understand better the ontogeny of CA systems formation, we analyzed wild-type embryos at stages from 1 to 5 dpf by anti-TH IHC. The first THir neurons arise between 18 and 20 hpf in the ventral diencephalon (Holzschuh et al.,^2001^) and soon project axons. At 24 hpf, the first THir longitudinal axons project from cells in the ventral diencephalon through the mid- and hindbrain into the spinal cord (Fig. 2A,B). These axons will pioneer the mlct. At this early stage, we could observe single projections with their growth cones still navigating loosely in the hindbrain (arrowhead in Fig. 2B).

At 24 hpf, the first noradrenergic THir neurons appear in the LC. Successively more dopaminergic THir cells arise in the diencephalon over the next day. Thus an increasing number of THir projections join the mlct. Soon the lateral CA projections (lcp) form caudal to the LC (arrowhead in Fig. 2C). The mlct axons leaving the diencephalic cluster (DC) project dorsally at first into the midbrain before they turn ventrally again to pass the LC and project through the hindbrain into the spinal cord (Fig. 2D). In the lateral view, the first projections from the diencephalon to the preoptic region become visible (preopticohypothalamic tract; poht in Fig. 2D).

After 2 days of development, the DA and NA neurons are still increasing in number. At 48 hpf, the mlct appears to be a thick fascicle, and the first transversal axons are crossing the midline in the most rostral hindbrain (arrows in Fig. 2E). The DA neurons in the preoptic region and the ventral diencephalon are now connected via the poht (Fig. 2F).

At 5 dpf, the overall pattern of nuclei and tracts in the developing zebrafish embryo is complete and corresponds well to that described for adult zebrafish (Ma,1994b,^1997^,^2003^). The difference between 3 dpf and 5 dpf appears mostly to correlate with an increase in the number of THir axons. Detailed analysis did not indicate that additional novel major tracts might have formed in 5 dpf larvae compared with 3 dpf (compare Fig. 1C–C′′,D with Fig. 2G,H). To allow use of gene knockdown by morpholinos and to facilitate further analysis of CA systems, we decided to conduct our experiments mainly in embryos at 3 dpf.

### The mlct does not navigate along the medial longitudinal fascicle early axonal scaffold

Early zebrafish neurons establish a stereotyped axonal scaffold of projections between 17 and 28 hpf (Chitnis and Kuwada,^1990^; Wilson et al.,^1990^), which has been proposed to serve as substrate for follower axons. One of the first axonal tracts evident in the developing embryo at 17 hpf is the medial longitudinal fascicle (mlf) emanating from the nucleus of medial longitudinal fascicle in the midbrain (Chitnis and Kuwada,^1990^) shortly before DA axonogenesis starts.

**Figure 3:**
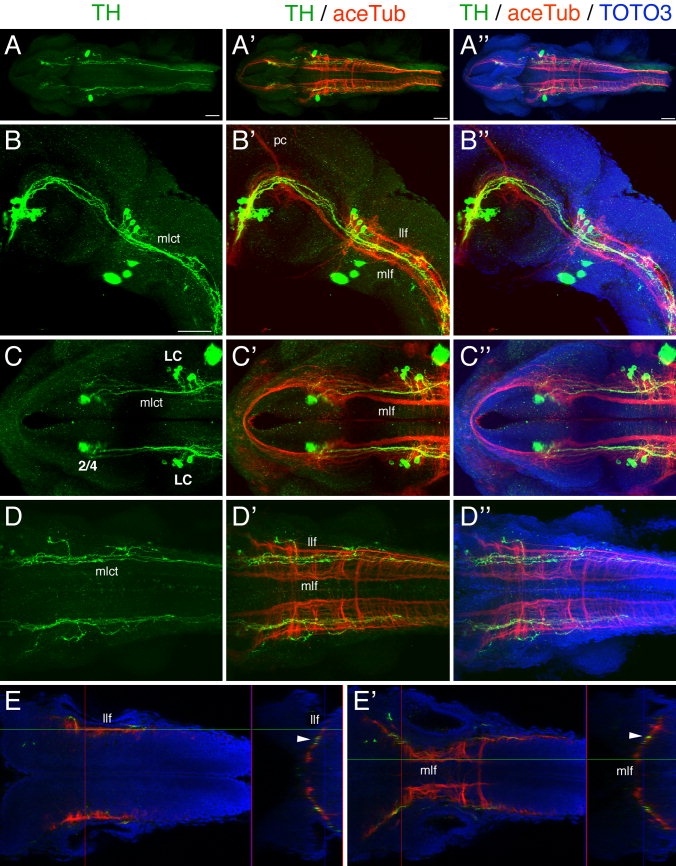
Longitudinal diencephalospinal THir projections do not colocalize with the mlf or llf of the primary axonal scaffold. We utilized antiacetylated tubulin (aceTub) in combination with anti-TH immunohistochemistry to investigate whether CA descending tracts follow the main primary axonal scaffolds characterized in zebrafish. Wild-type embryos were analyzed at 36 hpf and z-projections of confocal stacks of whole-mount immunohistochemistry combined with TOTO3 nuclear counterstaining prepared. Dorsal (A,C–E) and lateral (B) views, anterior to the left. **A–A′′:** Z-projection showing a dorsal overview. **B–B′′:** Lateral z-projection of diencephalon to hindbrain region. **C–C′′:** Dorsal z-projection of the brain region from the telencephalon to anterior hindbrain. **D–D′′:** Dorsal z-projection of hindbrain and spinal cord. The mlct does not project along the mlf or llf of the primary axonal scaffold. **E:** Single confocal dorsal plane showing the location of the llf in the hindbrain. The scan in z-direction shown on the right side reveals that mlct axons (arrowhead) do not correlate with the llf. **E′:** Single confocal dorsal plane showing the position of the mlf in the hindbrain. The scan in z-direction shown on the right side reveals that the mlct (arrowhead) does not correlate with the mlf. The numbering of ventral diencephalic DA groups is according to Rink and Wullimann (2002a). For abbreviations see list. A magenta green copy of this figure is available as Supporting Information Figure 2. Scale bars = 50 μm in A; 50 μm in B (applies to B–E′).

To analyze the position of CA tracts in relation to the early axonal scaffold, we used antiacetylated tubulin antibodies to visualize the axonal scaffold in combination with anti-TH IHC and TOTO-3 nuclear counterstaining in wild-type embryos at 36 hpf (dorsal overview, Fig. 3A–A′′). The lateral view (Fig. 3B–B′′) shows the mlf starting slightly caudal to the tract of the posterior commissure and projecting longitudinally into the hindbrain. Descending THir longitudinal axons projecting to the spinal cord do not join the mlf. Dorsal views on the fore- to hindbrain region (Fig. 3C–C′′) and hindbrain to spinal cord region (Fig. 3D–D′′) confirm the observation that longitudinal THir axons do not grow within the mlf but rather between the mlf and the lateral longitudinal fascicle (llf) at 36 hpf. Analysis of individual consecutive planes of a confocal stack and of confocal stack Z-sections reveals that the longitudinal THir tract is immunoreactive for acetylated tubulin, as are the majority of early axons (Fig. 3E,E′), but does not colocalize with the medial or lateral longitudinal fascicle. To distinguish these descending projections, we assigned the THir tract as mlct. Overall our data show that mlct axons grow in the vicinity of the mlf and llf but do not correlate with these early axonal scaffold tracts.

### Genetic dissection of dopaminergic and noradrenergic contributions

Some genetic mutations characterized in zebrafish make it possible to eliminate specific noradrenergic and/or dopaminergic cell groups, allowing analysis of their contributions to CA projections. Furthermore, morpholino gene knockdown in combination with such mutant zebrafish lines makes it possible to suppress multiple gene functions simultaneously. The CA phenotypes of the THir cell groups for the specific mutant and morpholino knockdown combinations are summarized schematically in Supporting Information Figure 5. We focused our analysis by anti-TH IHC on 3-day-old early larvae, because morpholino activity declines during the fourth and fifth days of development, and occasionally neurons form again.

**Figure 4:**
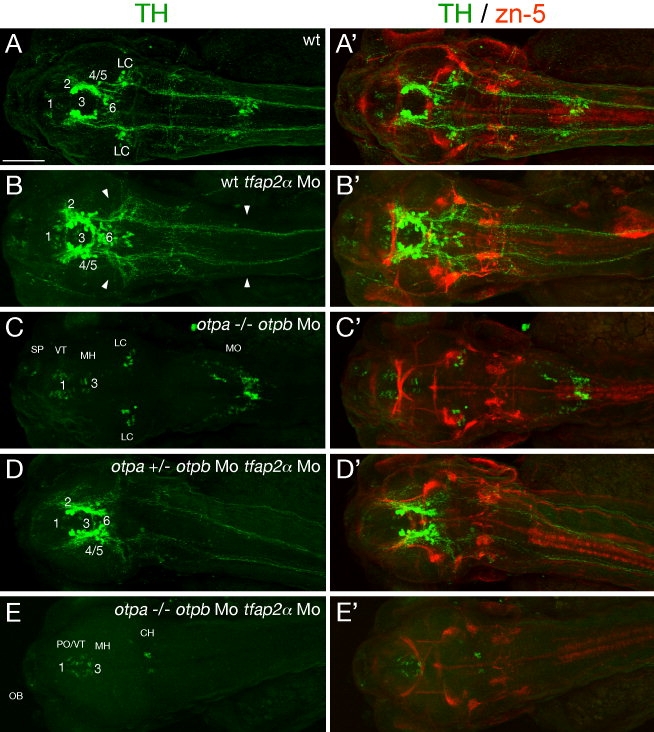
The majority of THir projections emanate from Otp-dependent diencephalic dopaminergic neurons. The relative contribution of DA and NA was investigated by genetic elimination of hindbrain NA or Otp-dependent DA neurons. Axonal projections were analyzed using anti-TH immunohistochemistry for CA tracts and zn-5 immunohistochemistry as a control for potential effects on general axonal tracts. Z-projections of confocal stacks of whole-mount 72 hpf embryos are shown; dorsal views, anterior to the left (A–E). **A,A′:** CA circuitry of wild-type control embryos. **B,B′:** Wild-type embryos injected with *tfap2α* morpholino (Mo) lack noradrenergic LC and MO neurons (arrowheads). **C,C′:** Homozygous *otpa*^–/–^ mutants injected with *otpb* Mo lack all Otp-dependent diencephalic DA neurons. **D,D′:** Heterozygous *otpa*^+/–^ embryos coinjected with *otpb* and *tfap2α* Mo lack noradrenergic LC and MO neurons but still develop most DA neurons, revealing that double Mo injection does not affect axonogenesis in general. **E,E′:** Homozygous *otpa*^–/–^ mutants coinjected with *otpb* and *tfap2α* Mo reveal the absence of brain NA and Otp-dependent DA neurons, whereas telencephalic, ventral thalamic, and medial hypothalamic DA neurons still form. Caudal hypothalamic CA neurons cannot consistently be detected at this stage (compare to C,D). A magenta green copy of this figure is available as Supporting Information Figure 3. The numbering of ventral diencephalic DA groups is according to Rink and Wullimann (2002a). For abbreviations see list. Scale bar = 100 μm.

First, we analyzed the noradrenergic contributions. The transcription factor *tfap2α* is required for expression of the NA phenotype in noradrenergic LC, MO, and AP neurons (Holzschuh et al.,2003a). In addition, the DA pretectal cluster and THir cells in the retina do not differentiate, in good correlation with the expression of *tfap2α* in these cells as opposed to the other DA groups without *tfap2α* expression. The THir phenotype induced by injection of *tfap2α* morpholino (Knight et al.,^2003^) into wild-type embryos reveals that the global pattern of CA tracts is similar to that of wild-type controls (Fig. 4A,B), although all NA neurons are missing.

So far, no single known mutation eliminates all DA neurons, but the two *otpa* and *otpb* genes are coordinately required for the development of the majority of posterior tubercular and hypothalamic DA neurons in zebrafish (Ryu et al.,^2007^). The remaining DA groups of the ventral thalamus (VT) and the most medial DA cells in the hypothalamus (MH), which are likely to be bipolar liquor-contacting group 3 DA neurons (Rink and Wullimann,2002b; Ryu et al.,^2007^), have been reported to form predominantly or exclusively local intradiencephalic projections (McLean and Fetcho,2004a). To eliminate both *otp* gene activities, we injected *otpb* morpholino into *otpa*^*m866*^ homozygous mutant embryos (Ryu et al.,^2007^). Normal differentiation of NA neurons in the hindbrain is not affected in *otpb* morpholino-injected *otpa*^*m866*^ mutant embryos. Anti-TH IHC showed that the global CA axonal network is strongly reduced in *otpa*^*m866*^ mutant embryos injected with *otpb* morpholino (Fig. 4C).

To analyze elimination of both NA and Otp-dependent DA projections, we coinjected *tfap2α* and *otpb* morpholinos into *otpa*^*m866*^ homozygous mutant embryos and, as a control, in heterozygous *otpa*^*m866*^ embryos. Double morpholino injections did not disturb the establishment of overall CA projections per se, because THir networks in heterozygous *otpa*^*m866*^ mutants injected with both morpholinos (Fig. 4D) are comparable to those of *tfap2α* morpholino-injected and wild-type embryos (Fig. 4B). In contrast, morpholino knockdown of *tfap2α* and *otpb* in homozygous *otpa*^*m866*^ mutants led to a massive reduction of CA projections (Fig. 4E), demonstrating that NA and Otp-dependent dopaminergic projections constitute together the vast majority of all far-projecting CA ascending and descending systems.

To control off-target effects of morpholinos, we visualized the well-described axonal projections stained by the zn-5 antibody (antineurolin/DM-GRASP; Trevarrow et al.,^1990^). The pattern of axons labeled by zn-5 antibody is comparable to that in wild-type embryos (Fig. 4A′) in all morpholino-injected embryos (Fig. 4B′–E′). The impact of the elimination of Tfap2a and Otp activity on the development of the THir cell groups and projections is summarized in Table 2 and Supplorting Information Figures 6 and 7 and will be analyzed in detail with regard to specific projections and tracts in the following sections.

**TABLE 2:** Summary of Phenotypes of Morpholino-Injected and/or Mutant Embryos^1^

Mutations	Morpholino	Affected CA groups	Affected CA projections	Figures
Wild type	—			1–6, 8
*otpa*^–/–^ (*m866*)	—	*otp*-positive diencephalic DA cells DC2, -4, -5, -6 (-)		—
*tfap2α*^–/–^ (*m819*)	—	LC (--; 0), MO (--; 0), Pr (--; 0)	act (-) prtep (0), mlp (0)	5
Wild type	*tfap2α*	LC (--; 0), MO (--; 0), Pr (--; 0)	act (-) prtep (0), mlp (0)	4, 7, 8
*otpa*^–/–^ (*m866*)	*otpb*	*otp*-positive DA cells (0) of posterior tuberculum and hypothalamus	poc (-), act (-), poht (--), mlct (--), lcp (0)	4, 7, 8
*otpa*^+/–^ (*m866*)	*otpb* and *tfap2α*	LC (--; 0), MO (--; 0), Pr (--; 0)	act (-) prtep (0), mlp (0)	4
*otpa*^–/–^ (*m866*)	*otpb* and *tfap2α*	LC (--; 0), MO (--; 0), Pr (--; 0), *otp*-positive diencephalic DA cells (0)	poc (-), act (0), poht (0), mlct (0), lcp (0), prtep (0), mlp (0)	4, 7, 8

1 Summary of the affected TH-ir cell groups and projections in the specific morpholino-injected and/or mutant embryos. Cell group or projection slightly reduced (-) strongly reduced (--), absent (0), or absent in most embryos except one or few cells in some embryos (--; 0).

### Dopaminergic systems in Tfap2α-deficient embryos

**Figure 5:**
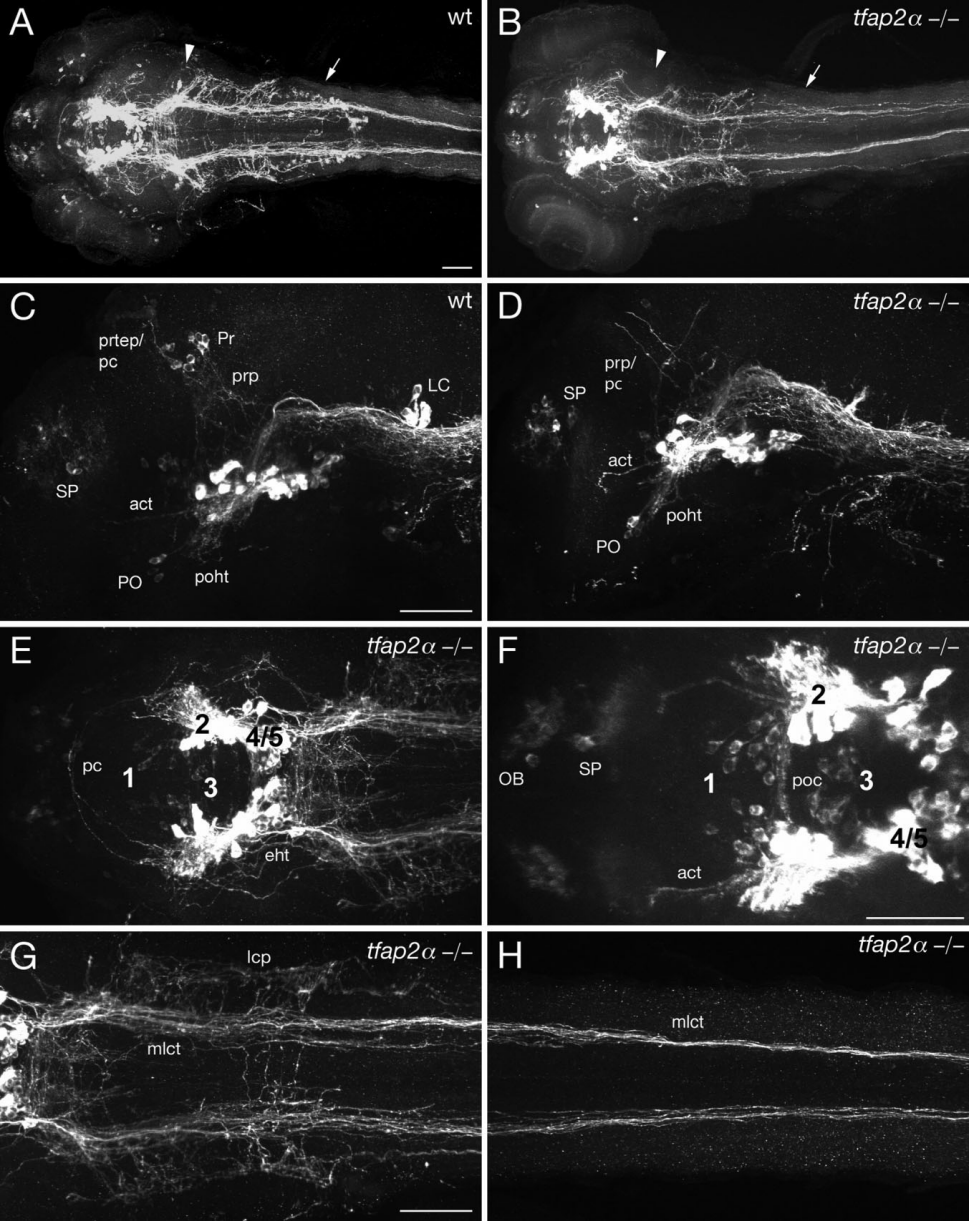
Comparison of CA projections in wild-type embryos and *tfap2α*^*m819*^ mutant embryos. Genetic elimination of noradrenergic LC and MO projections reveals DA contribution to CA projections. Z-projections of confocal stacks of whole-mount anti-TH immunohistochemistry in 72-hpf embryos are shown. Dorsal (A,B,E–H) and lateral (C,D) views, anterior to the left. **A,B:** Dorsal overviews of wild-type (A) and *tfap2α*^–/–^ mutant (B) embryos. The anatomical positions of the LC (arrowhead) and MO (arrow) are indicated. THir neurons of the LC and MO are not differentiated in *tfap2α*^–/–^ mutants. **C,D:** Lateral overviews of wild-type (C) and *tfap2α*^–/–^ (D) embryos. D: The THir pretectal cluster is missing in *tfap2α*^–/–^ embryos, but THir projections from the diencephalon to the pretectum (pc/prp) are visible. Ascending DA tracts (act) are also present in *tfap2α*^–/–^ mutants. **E:** Connectivity of diencephalic DA neurons in *tfap2α*^–/–^ mutants, dorsal part. The THir part of the pc is present. **F:** Connectivity of diencephalic DA neurons in *tfap2α*^–/–^ mutants, ventral part. The poc and act are shown. **G:** Hindbrain region of *tfap2α*^–/–^ mutants. Although NA neurons do not differentiate, hindbrain axonal projections are present (compare with Fig. 1D). **H:** Spinal cord region of *tfap2α*^–/–^ mutants. The mlct is comparable to that in wild-type embryos (see Fig. 1E). The numbering of ventral diencephalic DA groups is according to Rink and Wullimann (2002a). For abbreviations see list. Scale bars = 50 μm in A (applies to A,B); 50 μm in C (applies to C–E); 50 μm in F; 50 μm in G (applies to G,H).

**Figure 6:**
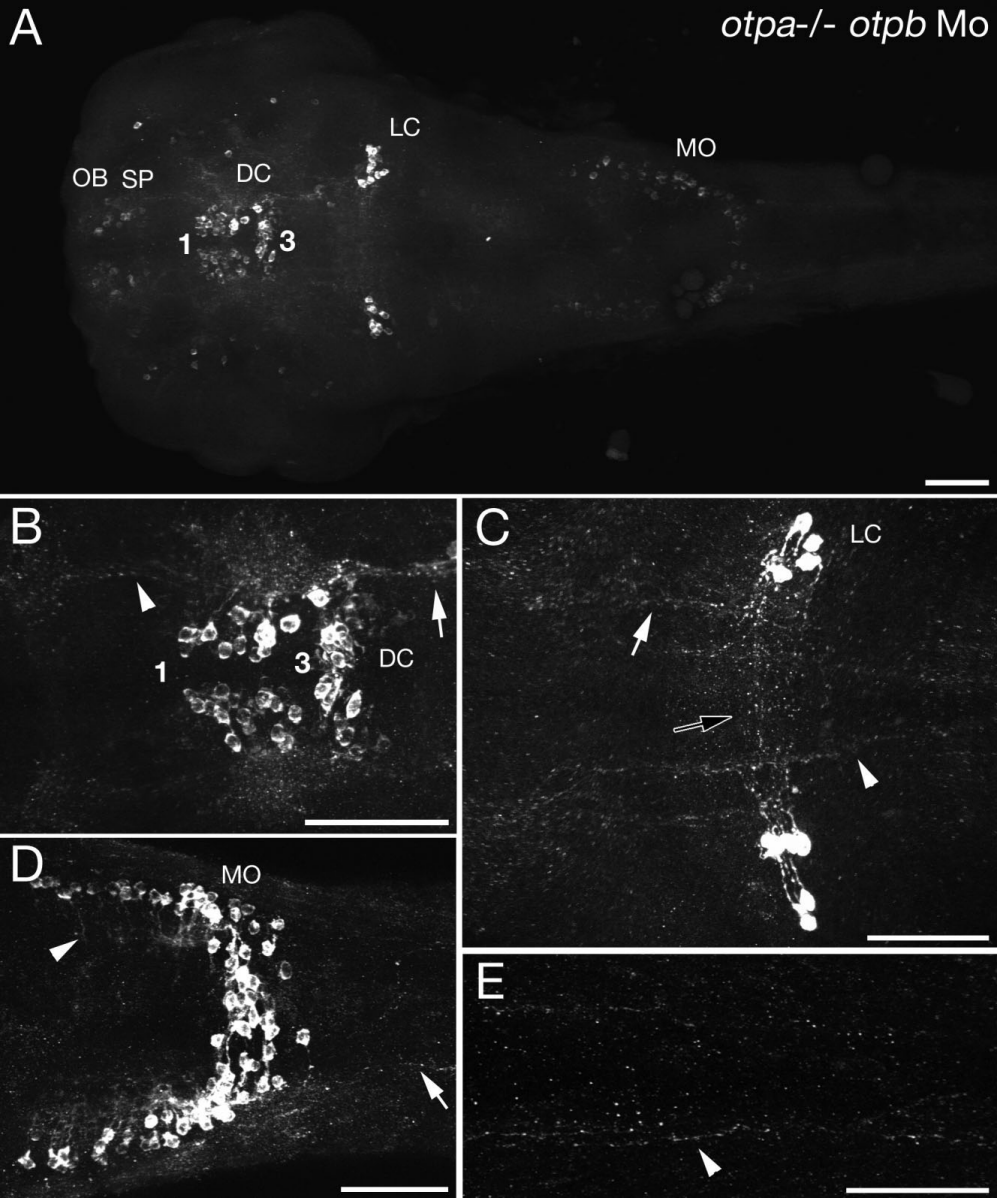
Comparison of CA projections in wild-type embryos and embryos completely devoid of Otp-specified DA neurons. A–E: Activity of both *otpa* and *otpb* genes was eliminated by injection of 2 ng *otpb* morpholinos into *otpa*^*m866*^ homozygous mutant embryos. Z-projections of confocal stacks of whole-mount anti-TH immunohistochemistry in 72-hpf embryos are shown; dorsal views, anterior to the left. **A:** Embryos deficient for all Otp activity do not develop posterior tubercular (groups 2 and 4) or lateral hypothalamic (groups 5 and 6) DA neuronal groups (for numbering of groups, see Fig. 9 and Supp. Info. Fig 1), whereas NA neurons (LC, MO) and Otp-independent DA neurons (OB, SP, PO, DC for ventral thalamic and medial as well as caudal hypothalamic groups) are present. The number of CA projections is drastically reduced. **B:** Axonal fibers are detectable in the diencephalon, including ascending projections to the telencephalon (arrowhead) and projections into the posterior hypothalamus (arrow). **C:** THir projections in the mid- and hindbrain: anterior mlct (white arrow), commissural (black arrow), and posterior mlct (arrowhead) projections in the region of the noradrenergic LC. **D:** In the MO, fine axonal projections take a medioventral route (arrowhead). A small number of very thin longitudinal projections can be detected in the hindbrain spinal cord, posterior to the MO NA neurons. **E:** THir longitudinal projections in the spinal cord (arrowhead). The numbering of ventral diencephalic DA groups is according to Rink and Wullimann (2002a). For abbreviations see list. Scale bars = 50 μm.

We used *tfap2α*^*m819*^ mutant embryos to visualize DA circuitry in the absence of NA neurons, because the NA phenotype of the *tfap2α* morpholino-injected embryos was not completely penetrant (Supp. Info. Fig. 6). THir NA neurons in *tfap2α*^*m819*^ mutant embryos do not differentiate until 4 dpf (Holzschuh et al.,2003b). Therefore, we can consider all THir projections to be DA in *tfap2α*^*m819*^ mutants at 3 dpf. THir circuitry in *tfap2α*^*m819*^ mutant embryos largely resembles the wild-type CA scaffold, despite the loss of NA fibers (compare Fig. 5A with B), confirming the observations we made in *tfap2α* morpholino-injected embryos (Fig. 4B).

A detailed analysis of the forebrain demonstrates that the main THir projections are present in *tfap2α*^*m819*^ mutants (Fig. 5D–F) compared with wild-type embryos (Fig. 5C). This includes the poht (Fig. 5D), pc, eht (Fig. 5E), and poc (Fig. 5F). In wild-type embryos, a connection of the pretectal DA neurons with ventral brain regions (prp) and THir axons growing dorsally from the pretectal cluster to the tectum (prtep) can be distinguished (Fig. 5C). Although the pretectal DA cells are missing in *tfap2α*^*m819*^ mutants, we observed THir projections into the pretectum (Fig. 5D), which demonstrates that the THir prp have a prominent contribution of dorsally projecting DA fibers. Furthermore, we could find in all *tfap2α*^*m819*^ mutants analyzed (n = 12) THir fibers projecting into the subpallium (Fig. 5D,F). This observation reveals ascending DA contributions to the anterior CA tract.

Analysis of the *tfap2α*^*m819*^ mutant hindbrain clearly demonstrates the lack of all NA contributions to THir circuitry in mutant embryos. Nevertheless, the typical THir axonal scaffold in the hindbrain and spinal cord is still present (Fig. 5G,H), and the quantity and quality of projections are comparable to those of the wild-type mlct and lcp, respectively, with regard to thickness and fasciculation (compare with Fig. 1D,E). In summary, these data reveal a predominantly DA contribution to THir projections in forebrain, hindbrain, and spinal cord.

### Otp-dependent dopaminergic neurons in the diencephalon constitute the majority of descending dopaminergic projections

A more detailed analysis of *otpa*- and *otpb*-deficient embryos confirmed absence of the majority of DA neurons of the posterior tuberculum and hypothalamus and of their axonal projections (Fig. 6A). With the assumption that the remaining DA groups of olfactory bulb, subpallium, preoptic, and medial and caudal hypothalamic regions are predominantly projecting locally, similarly to amniote vertebrates (Smeets and González,^2000^), the few dorsally projecting as well as descending longitudinal THir fibers found in these embryos are thus probably of NA origin.

Higher magnification revealed THir axons between the tel- and diencephalon, corresponding to the act in Otp-deficient embryos (Fig. 6B). Other longitudinal THir fibers are running through the ventral diencephalon, midbrain, and hindbrain rostral to the LC (Fig. 6B,C), probably emanating from the NA LC neurons. The bilateral THir LC clusters appear to be connected across the midline via transversal tracts (Fig. 6C). A small number of very thin THir projections was found caudal to the LC, passing the MO and projecting into the spinal cord (Fig. 6D,E). Noradrenergic MO cells have fine axonal projections, which take a medial–ventral route. In summary, these findings suggest that ascending and descending THir projections originate predominantly from Otp-dependent DA neurons of the posterior tuberculum and hypothalamus.

### The anterior catecholaminergic tract

**Figure 7:**
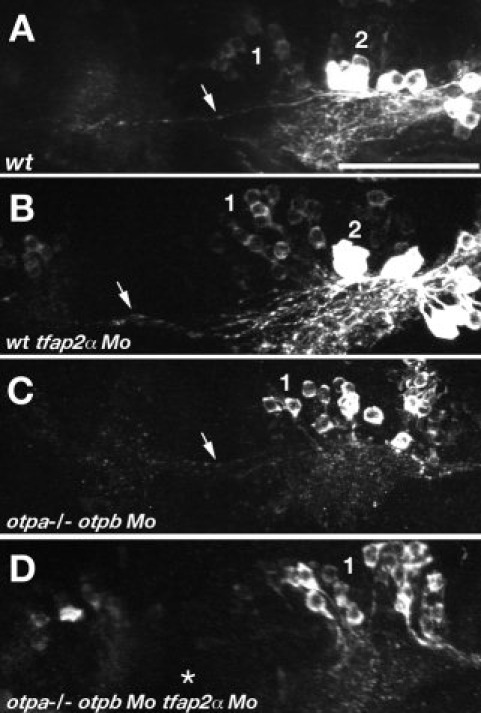
DA and NA contributions to the act. Genetic elimination of NA or Otp-dependent DA THir projections reveals ascending contributions. Z-projections of confocal stacks of whole-mount anti-TH immunohistochemistry in 72-hpf embryos are shown, dorsal views, anterior to the left (A–D). The act (arrows) axonal projections of wild-type embryos (**A**), wild-type embryos injected with *tfap2α* Mo (**B**), *otpa*^*m866*^ mutant embryos injected with *otpb* Mo (**C**), and *otpa*^*m866*^ mutant embryos coinjected with *otpb* and *tfap2α* Mo (**D**) were analyzed. *tfap2α* Mo-injected wild-type embryos (arrow in B) and *otpb* Mo-injected *otpa*^*m866*^ mutant embryos (arrow in C) both have THir projections between the diencephalic cluster and subpallium. THir act axons are not detectable in *otpa*^*m866*^ mutant embryos coinjected with *otpb* and *tfap2α* Mo (asterisk in D). The numbering of ventral diencephalic DA groups is according to Rink and Wullimann (2002a). Scale bar = 50 μm.

Given the importance of long ascending and descending CA projections for motor behavior, we decided to discriminate more precisely between NA and DA contributions to two major pathways, the act and the mlct. The axons contributing to the act establish an axonal CA connection between the ventral diencephalon and the subpallium (Fig. 7A). Axons of the act are still present both in *tfap2α* morpholino-injected embryos lacking noradrenergic THir neurons (Fig. 7B; see also Fig. 5D,F) and in *otpb* morpholino-injected *otpa*^*m866*^ homozygous embryos lacking contributions of Otp-dependent DA neurons (Fig. 7C). However, act axons were not detected in *otpa*^*m866*^ mutants coinjected with both *otpb* and *tfap2α* morpholinos (Fig. 7D). The latter embryos have no DA projections of *otp*-expressing neurons, nor do they form noradrenergic THir axons. Therefore, axons from Otp-independent DA cells of the ventral thalamus and medial hypothalamus do not appear to contribute to the anterior CA tract, insofar as these neurons are still present in *otpa*^*m866*^ mutants injected with *otpb* morpholino. Inasmuch as the anterior CA tract is lost in *otpa*^*m866*^-deficient embryos injected with both *otpb* and *tfap2α* morpholinos in contrast to *otpa*^*m866*^ homozygous embryos injected only with *otpb* morpholino, there must be NA contributions to this tract. Our data thus reveal that the act has contributions from both NA projections emanating from the hindbrain and DA projections of the ventral DA neurons. During later development, other CA projections may also contribute to the act.

### The medial longitudinal catecholaminergic tract

**Figure 8:**
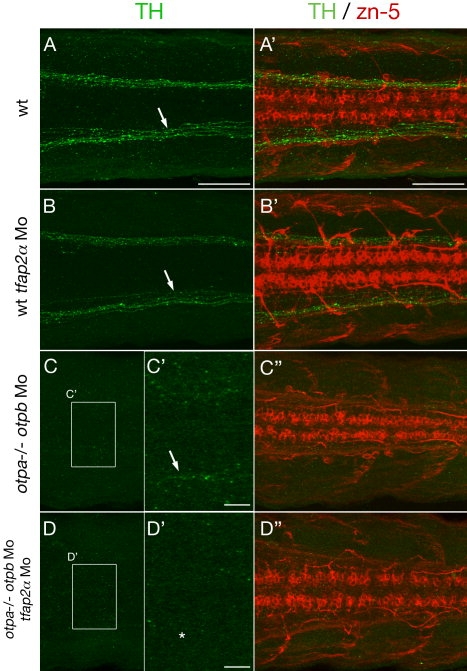
DA and NA contributions to longitudinal spinal THir tracts. Genetic elimination of NA or Otp-dependent DA THir projections reveals spinal CA contributions. Z-projections of confocal stacks of whole-mount anti-TH and zn-5 coimmunohistochemistry in 72-hpf embryos are shown; dorsal views, anterior to the left (A–D). The mlct (arrows) axonal projections of wild-type embryos (**A**), wild-type embryos injected with *tfap2α* Mo (**B**), *otpa*^*m866*^ mutant embryos injected with *otpb* Mo (**C**), and *otpa*^*m866*^ mutant embryos coinjected with *otpb* and *tfap2α* Mo (**D**) were analyzed in the trunk part of the spinal cord. There are no obvious changes of the mlct in embryos injected with *tfap2α* Mo (arrow in B) compared with wild type (arrow in A). The injection of *otpb* Mo in *otpa*^*m866*^ mutant embryos reduces the axons to one or two visible axonal fibers (arrow in C′; C′ is a ×2.5 magnification of C). No THir axons are detectable in embryos devoid of Otp and Tfap2α activity (asterisk in D′; D′ is a ×2.5 magnification of D). As a control, axons labeled by zn-5 antibody are not affected (A′,B′,C′′,D′′). A magenta green copy of this figure is available as Supporting Information Figure 4. Scale bars = 50 μm in A (applies to A–D); 50 μm in A′ (applies to A′,B′,C′′,D′′); 10 μm in C′,D′.

We next analyzed in more detail the mlct, which is the most prominent tract in the zebrafish CA system connecting the forebrain with the hindbrain and spinal cord. In the wild type, the mlct is fasciculated in the ventrolateral spinal cord and consists of many THir axons (Fig. 8A). The spinal mlct is largely unchanged in *tfap2α*-deficient embryos lacking THir NA neurons (Fig. 8B). In contrast, elimination of Otp activity by morpholino knockdown of *otpb* in *otpa*^*m866*^ mutants led to a severe reduction of THir longitudinal fibers in the spinal cord, which were only faintly detectable at high magnifications (Fig. 8C; see also inset C′). Coinjection of *tfap2α* and *otpb* morpholinos in *otpa*^*m866*^ homozygous embryos caused complete loss of THir mlct projections (Fig. 8D,D′). Colabeling with zn-5 antibody reveals normal development of secondary motorneurons and their axons in the spinal cord in all morpholino-injected embryos (Fig. 8B′,C′′,D′′) compared with wild-type embryos (Fig. 8A′), demonstrating that the observed changes in CA systems are not caused by general defects. We therefore conclude that Otp-dependent DA neurons of the posterior tuberculum and lateral hypothalamus provide the predominant contribution to descending mlct projections.

## DISCUSSION

**Figure 9:**
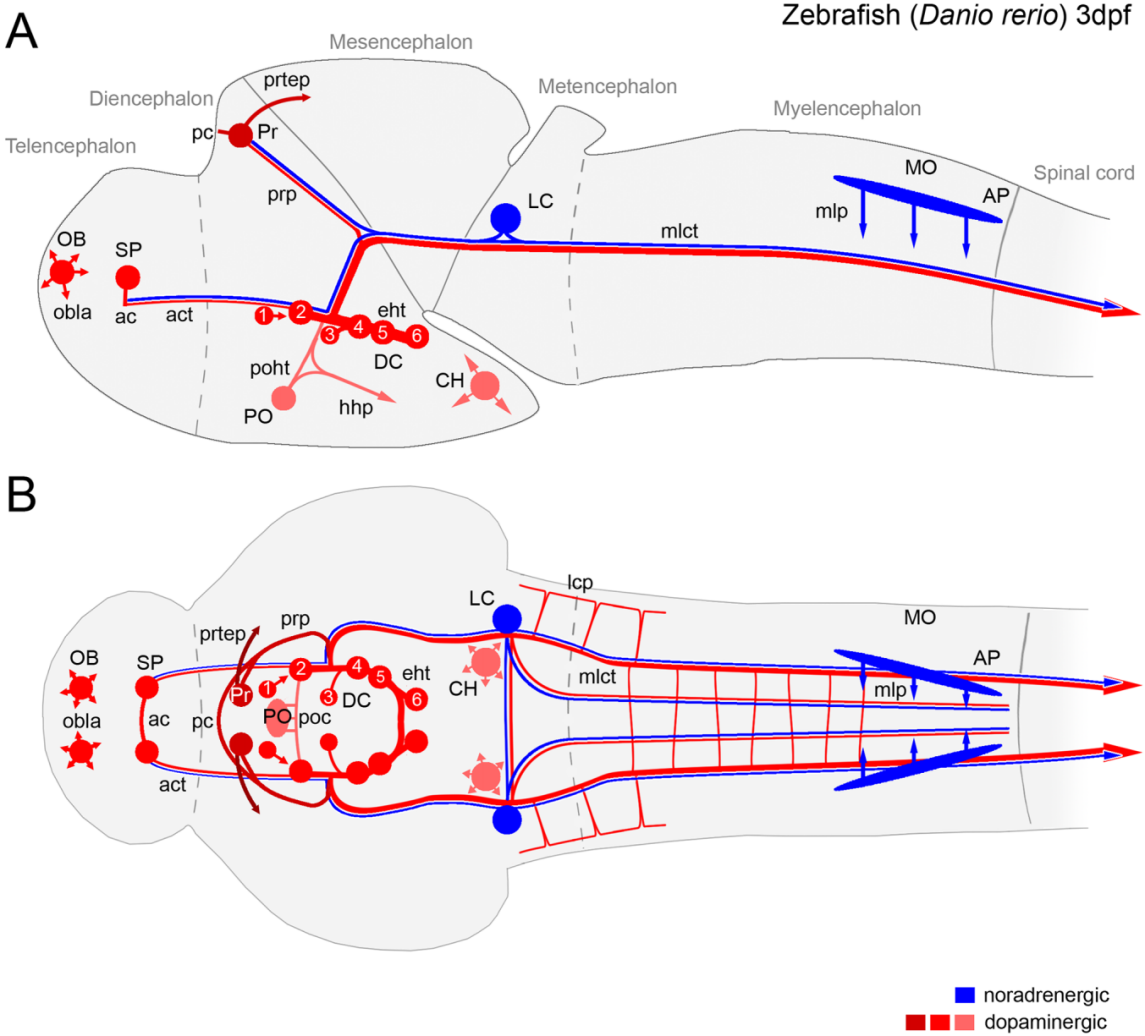
Schematic overview of DA and NA projections in 3-day-old zebrafish larvae. Schematic representation of major THir projection paths in correlation with anatomical location of DA (red) and NA (blue) somata summarized for the CNS of 3-dpf zebrafish larvae. Lateral (**A**) and dorsal (**B**) overviews. The relative dorsoventral position of the DA cells is indicated by different brightnesses, from dark red (dorsal) to light red (ventral). The numbering of ventral diencephalic DA groups is according to Rink and Wullimann (2002a): DC1, ventral thalamic DA neurons; DC2 and -4, rostral and caudal posterior tubercular DA neurons; DC3, medial hypothalamic DA neurons (liquor contacting); DC5 and -6, hypothalamic DA groups; DC7 is not listed here but correlates with the caudal hypothalamic groups shown here. The medial DA and NA projections in the hindbrain area represent individual axons observed at variable mediolateral positions (see Fig. 2H).

Although dopamine systems have been intensely studied in many vertebrates based on their disease relevance, the complex contributions of NA systems to basic physiology (Björklund and Dunnett, 2007; Iversen and Iversen,^2007^), sleep-wake and arousal state (Ordway et al.,^2007^), cognition (Sara,^2009^), and motor control (Jordan et al.,^2008^) as well as links to the dopamine system and Parkinson's disease (Rommelfanger and Weinshenker,^2007^) have had less attention. Although the anatomy of both CA systems has been analyzed in detail (Smeets and González,^2000^), the relative DA and NA contributions to the developing CA systems are not well known, especially for anamniote vertebrate model organisms. Here, we have analyzed the pattern of CA axonal projections in 1–5-day-old zebrafish embryos and early larvae. Our findings extend previous studies (Ma,1994b,^1997^; Kaslin and Panula,^2001^; Rink and Wullimann,^2001^,2002a; McLean and Fetcho,2004b; Sallinen et al.,^2009^) in that we differentiate between contributions of different DA and NA groups to CA connectivity by utilizing genetic tools. The major projection paths relative to the anatomical location of DA and NA somata in the brain of 3-day-old zebrafish are summarized in Figure 9. The anatomical locations of CA tracts visualized here for larval zebrafish match those described in previous studies (Rink and Wullimann,2002a; McLean and Fetcho,2004a). However, our experiments focus on visualizing THir circuitry in whole-mount embryos utilizing confocal microscopy, thereby providing an overview of CA connections in the anatomical context of the embryo.

Mechanisms for axonal pathfinding and establishment of CA tracts in zebrafish have not been described so far. The axonal scaffold hypothesis proposes that axons of later-born neurons may often grow along an initial set of projections, using them as an axonal scaffold (Chitnis and Kuwada,^1990^; Wilson et al.,^1990^). However, we have shown here that the mlct tract does not correlate with the two most prominent longitudinal fascicles labeled by antiacetylated tubulin antibody. Therefore, positioning of CA tracts appears to be determined independently of the earlier-developing scaffold of other axonal tracts, such as the mlf or llf. It is possible that the first longitudinal THir axons emanating from the diencephalon between 20 and 24 hpf function themselves as pioneer axons to guide those that follow, as has been reported for axons of retinal ganglion cells (Pittman et al.,^2008^). Molecular cues such as slits, netrins, semaphorins, or ephrins provided by the environment could play important roles for the pathfinding of CA projections (Van den Heuvel and Pasterkamp,^2008^).

A major focus of our work was the identification of neuronal subset-specific connectivity in the CA systems and elucidating relative contributions of NA and DA projections. A previous study examined NA systems in adult zebrafish by using an antibody against dopamine beta hydroxylase that was detected within the cell body and accompanying processes of NA neurons (Kaslin and Panula,^2001^). In our hands, this antibody did not work as well in larval zebrafish and did not stain axons efficiently (data not shown), which has also been reported by McLean and Fetcho (2004a), so immunohistochemistry was not available to distinguish DA and NA neurons in the developing brain. In contrast, the zebrafish-specific anti-TH antibody that we made (Ryu et al.,^2007^) visualizes CA axons very well. To distinguish contributions of CA neuronal groups, we therefore depleted defined CA subgroups by using genetics or morpholino knockdown of transcription factors important for the development of specific neuronal groups.

Analyses of THir circuitry in *tfap2α*^*m819*^ mutants and embryos injected with *tfap2α* morpholino both indicate that DA projections provide by far the dominant portion of CA axon tracts. Morpholino knockdown of *otpb* in *otpa*^*m866*^ mutant embryos further refined this finding by showing that the majority of far-projecting DA axons originates from Otp-dependent neurons of the posterior tuberculum and hypothalamus. Previous work from our group suggested that a relatively large portion of the DA systems was under the control of Otp activity in zebrafish compared with mammals, where the A11 group is Otp dependent (Ryu et al.,^2007^). Our data also reveal similarities in projection behavior of Otp-dependent neurons in zebrafish and A11 group in mammals: both have predominant caudal projections into the spinal cord (Björklund and Skagerberg,^1979^) but also ascending projections to the telencephalon (Takada et al.,^1988^). Consistently with our observation of a DA diencephalospinal tract, retrograde labeling in zebrafish larvae has previously demonstrated that the source of spinal TH immunoreactivity is in the posterior tuberculum and that THir neurons might interact with primary motor neurons (McLean and Fetcho,2004b). Similar studies in amphibian species have demonstrated the contribution of THir cells of the posterior tuberculum and LC to the CA innervation of the spinal cord (Sanchez-Camacho et al.,^2002^). Here, we succeeded in genetically distinguishing between the more prominent DA and the rather minor NA contributions to the mlct connecting the ventral diencephalon to the spinal cord. The relevance of this proportional dominance of the diencephalospinal DA system for zebrafish behavior remains to be determined, because the function of the diencephalospinal Otp-dependent A11-like DA tract in vertebrates is still elusive. For mammals, the A11 contribution to spinal somatosensory and somatomotor circuits has been discussed (Jordan et al.,^2008^), and potential links to diseases such as restless legs (Ekbom) syndrome have been proposed (Clemens et al.,^2006^). It is possible that an early control of sensorimotor activity is important for the rapidly developing zebrafish larvae, isofar as they are already efficient hunters after 4–5 days of development (Budick and O'Malley,^2000^).

We could demonstrate both NA and DA contributions to the act linking diencephalon and ventral telencephalon. In mammals, the most prominent DA projections are emanating from midbrain DA neurons and ascend along different pathways into the striatal, cortical, and limbic areas (Björklund and Dunnett, 2007). For zebrafish, dye-tracing experiments in adult animals indicated that an ascending DA system projects from the posterior tuberculum to the subpallium in zebrafish (Rink and Wullimann,^2001^,2002a). Our study in 3-day-old larvae indicates that such ascending DA projections already form during early developmental stages. Together with the results obtained in the dye-tracing experiments mentioned above (Rink and Wullimann,2002a), our data indicate that ascending DA projections originate from Otp-dependent DA cells of the posterior tuberculum. Previous studies in fish failed to demonstrate DA projections from the hypothalamus to the telencephalon (Rink and Wullimann,^2001^,^2004^), so we consider it unlikely that the Otp-dependent hypothalamic DA cells also contribute to the ascending DA projections. However, connectivity between telencephlon and hypothalamus in teleosts has been well established (Folgueira et al.,2004a,2004b; Yamamoto and Ito,^2008^), such that further experiments will be needed to exclude ascending DA contributions from the hypothalamus. Although mammals have A11 ascending as well as nigrostriatal ascending systems, work on lamprey has led to the hypothesis that striatal connectivity of the paratubercular region in lamprey may fulfill functions similar to those of ascending projections of the mammalian substantia nigra (Pombal and Puelles,^1999^).

In the present study, we also detected ascending NA projections in larval zebrafish. Tracer studies in adult zebrafish revealed that the pallium and subpallium receive NA input from the LC (Rink and Wullimann,^2004^). Extensive telencephalic NA targets have been reported for mammals (Sara,^2009^). Up to 40% of the noradrenergic LC neurons project to the olfactory bulb in rats (Shipley et al.,^1985^).

In summary, we found that DA neurons of the posterior tuberculum constitute the majority of descending CA axons into hindbrain and spinal cord and supply at the same time ascending projections to the ventral telencephalon. NA neurons also project into hindbrain and spinal cord and show ascending projections to the ventral telencephalon. Our work also has certain limitations. The genetic approach forced us to limit our analysis to early larval stages up to 3 days of development, which might have led us to miss some projections that form later. The projection behavior of specific DA subgroups or even individual neurons in the posterior tuberculum and hypothalamus needs further analysis. A refined mapping of their connections would be helpful to define DA circuitry and to reveal potential homologies to amniote vertebrates, ultimately contributing to an understanding of the evolution of dopaminergic systems. However, our genetic analysis suggests that the Otp-dependent, A11-related ventral diencephalic diencephalospinal and ascending DA systems may have evolved as major modulators of motor behavior and potentially also general behavior patterns in early vertebrates.

## Abbreviations

AAN: arch associated NA cluster
ac: anterior commissure
acla: amacrine cells local arbors
act: anterior CA tract
AP: area postrema
ccp: cerebellar CA projection
CH: caudal hypothalamus
DC: diencephalic cluster
eht: endohypothalamic tract
hhp: hypothalamic–hypophyseal projections
LC: locus coeruleus
lcp: lateral CA projections
llf: lateral longitudinal fascicle
mlct: medial longitudinal CA tract
mlf: medial longitudinal fascicle
mlp: medulla oblongata local projections
MH: medial hypothalamus
MO: medulla oblongata
Mo: morpholino antisense oligonucleotide
OB: olfactory bulb
obla: olfactory bulb local arbors
pc: posterior commissure
PO: preoptic region
poc: postoptic commissure
poht: preopticohypothalamic tract
Pr: pretectum
prp: pretectal projections
prtep: pretectotectal projections
SC: spinal cord
SP: subpallium
VT: ventral thalamus
