# Supplementary figures and images for: Genetic dissection of dopaminergic and noradrenergic contributions to catecholaminergic tracts in early larval zebrafish
Source: J Comp Neurol. 2009 Aug 16;518(4):439–58. doi: 10.1002/cne.22214 (PMC2841826; doi:10.1002/cne.22214)

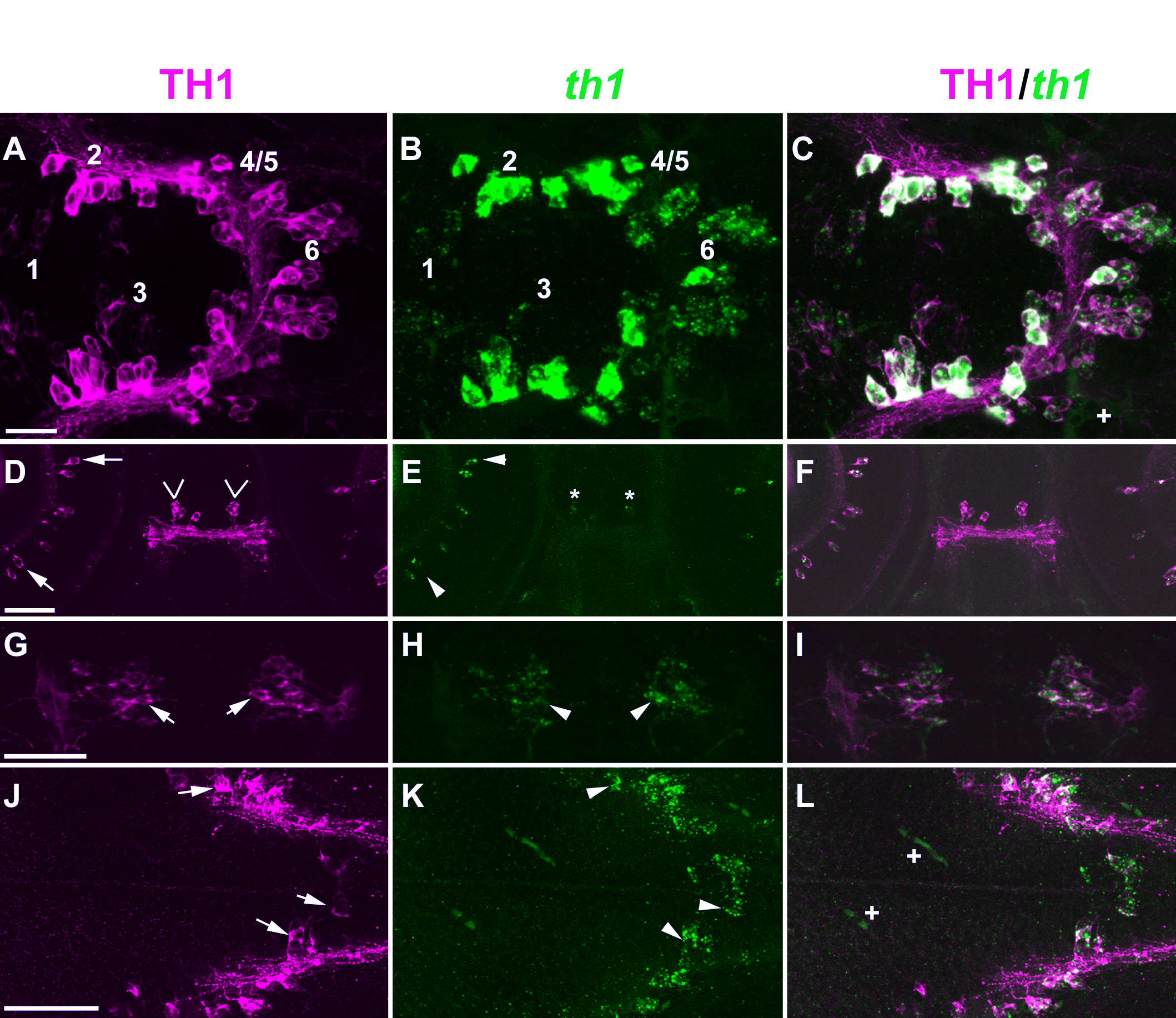

Supplement: Supplementary file 1 [file cne0518-0439-SD1.tif]

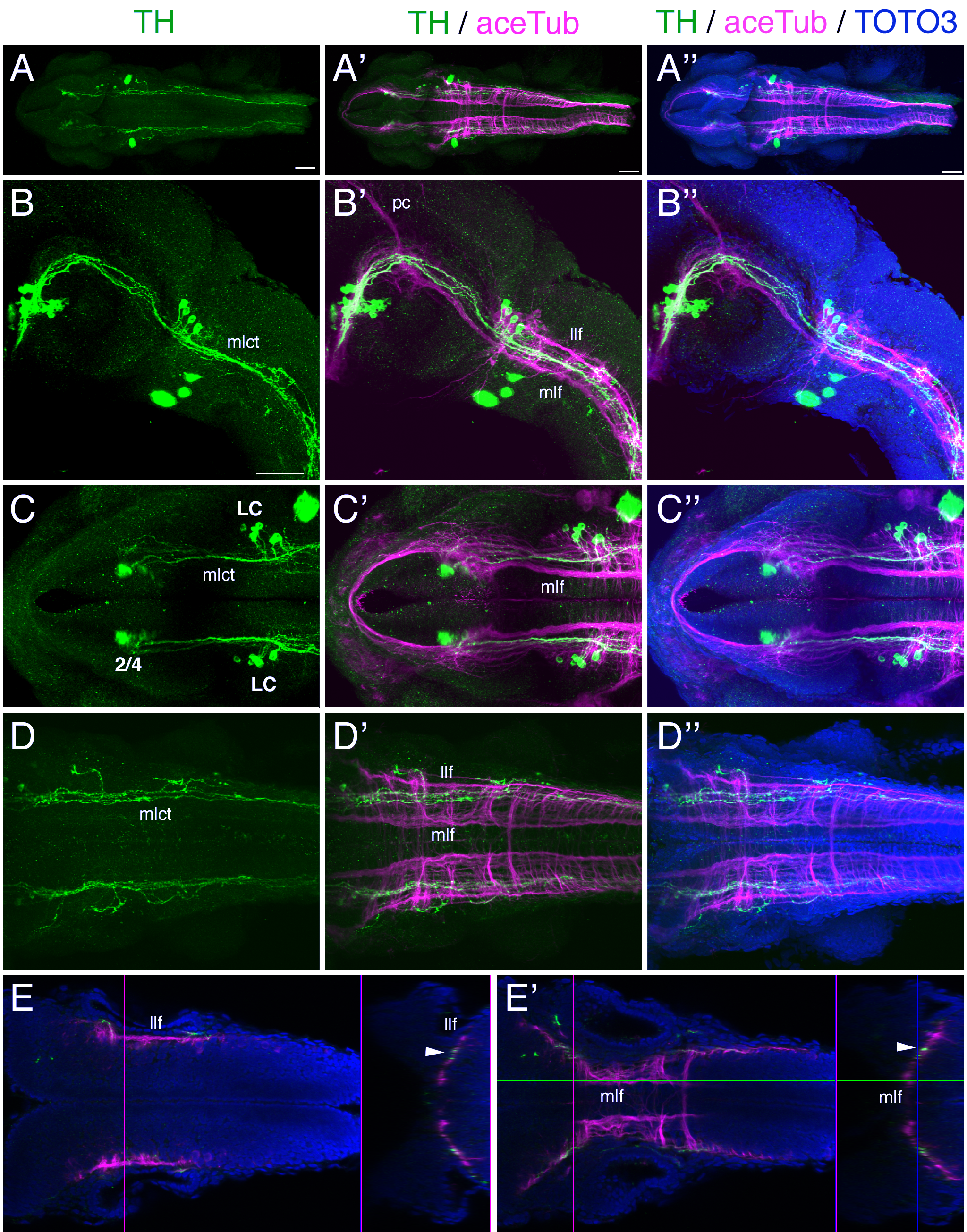

Supplement: Supplementary file 2 [file cne0518-0439-SD2.tif]

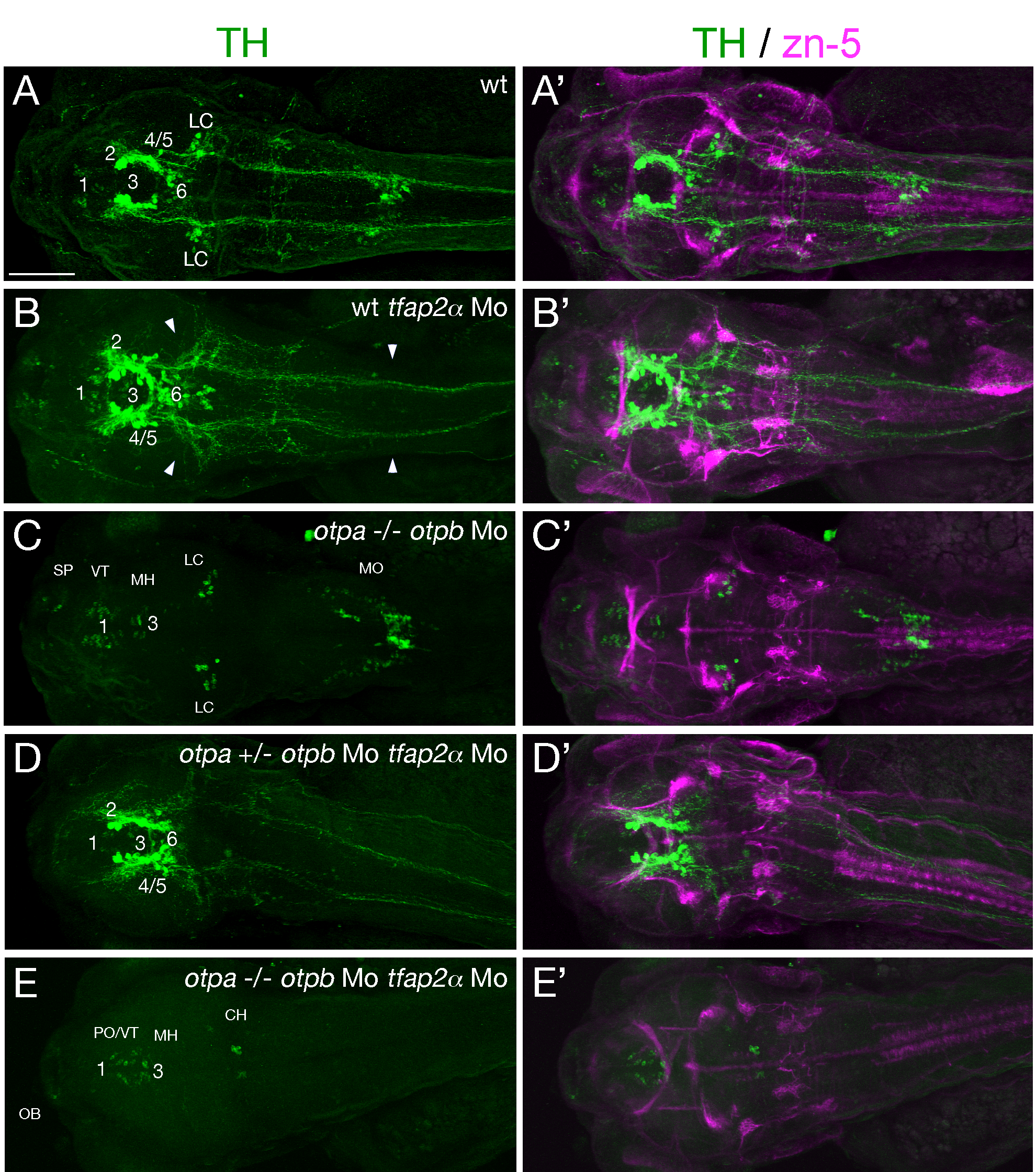

Supplement: Supplementary file 3 [file cne0518-0439-SD3.tif]

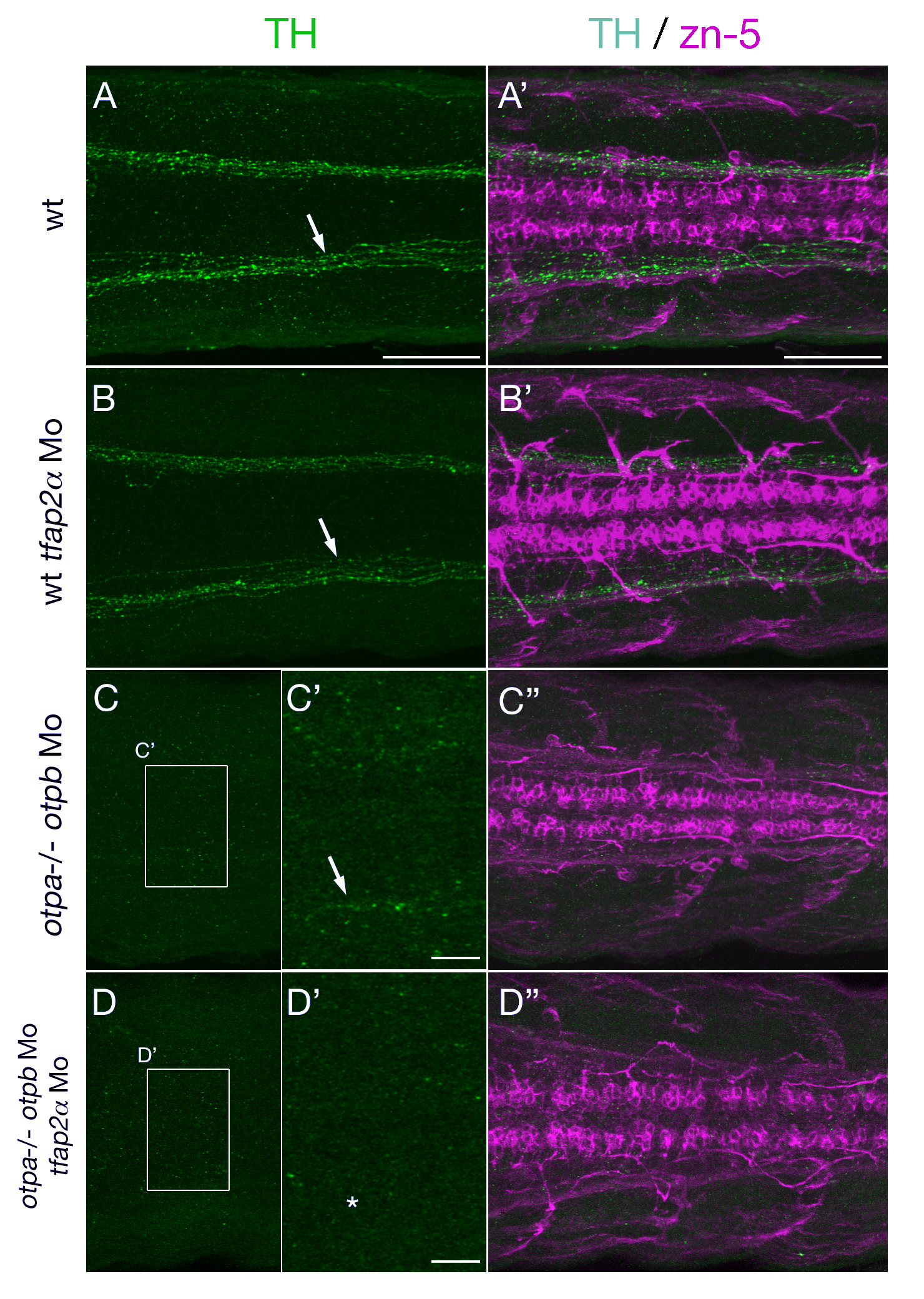

Supplement: Supplementary file 4 [file cne0518-0439-SD4.tif]

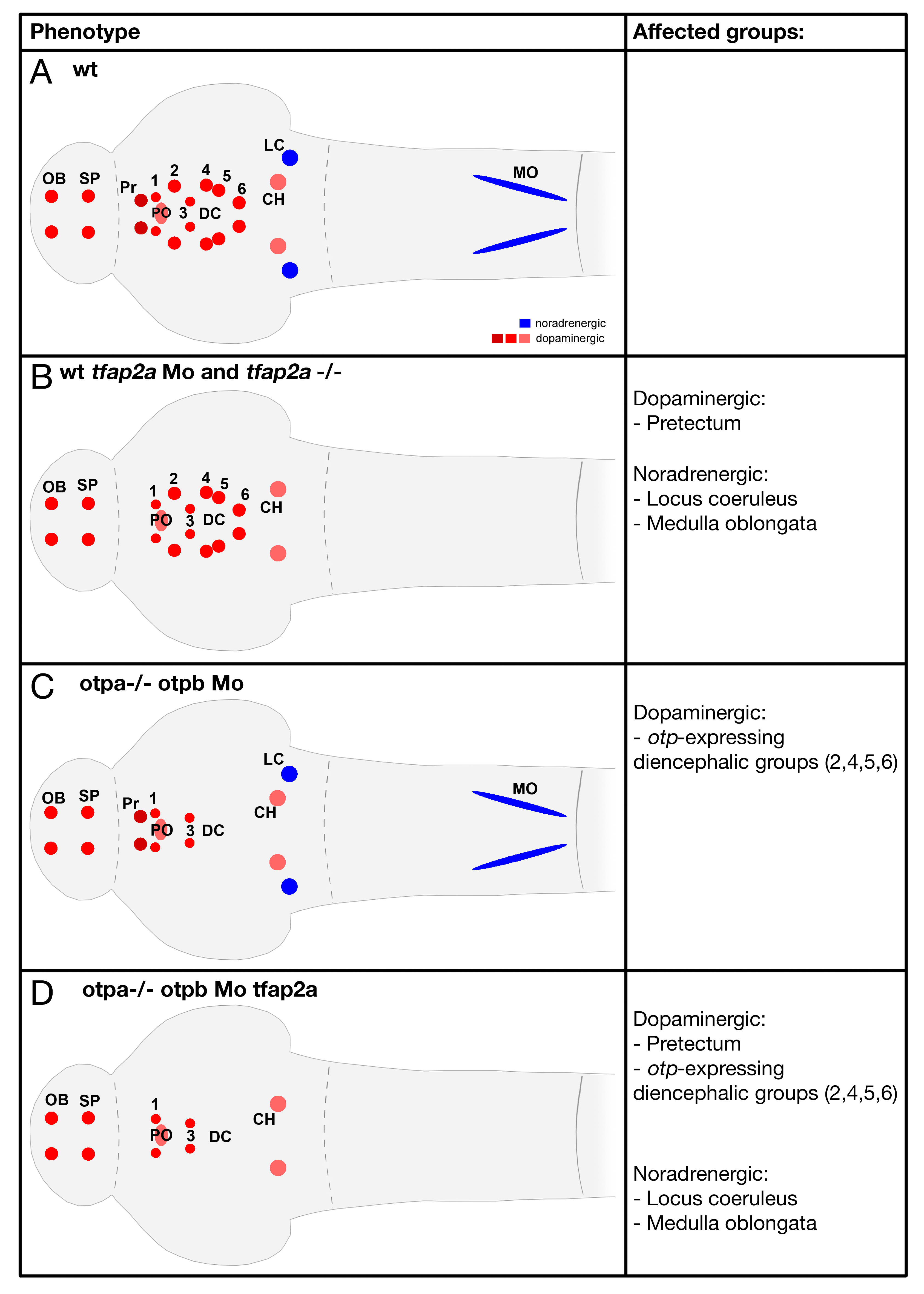

Supplement: Supplementary file 5 [file cne0518-0439-SD5.tif]

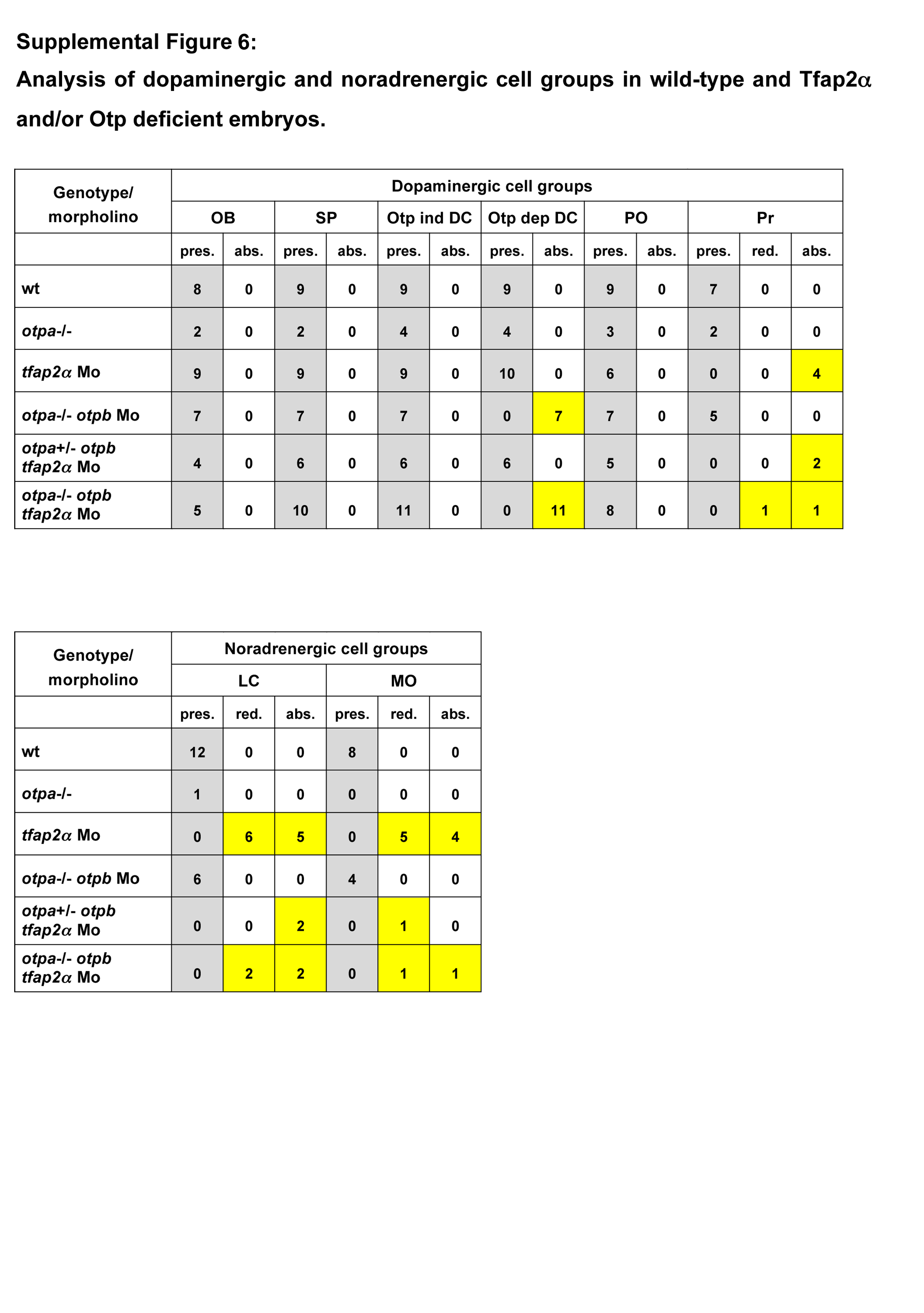

Supplement: Supplementary file 6 [file cne0518-0439-SD6.tif]

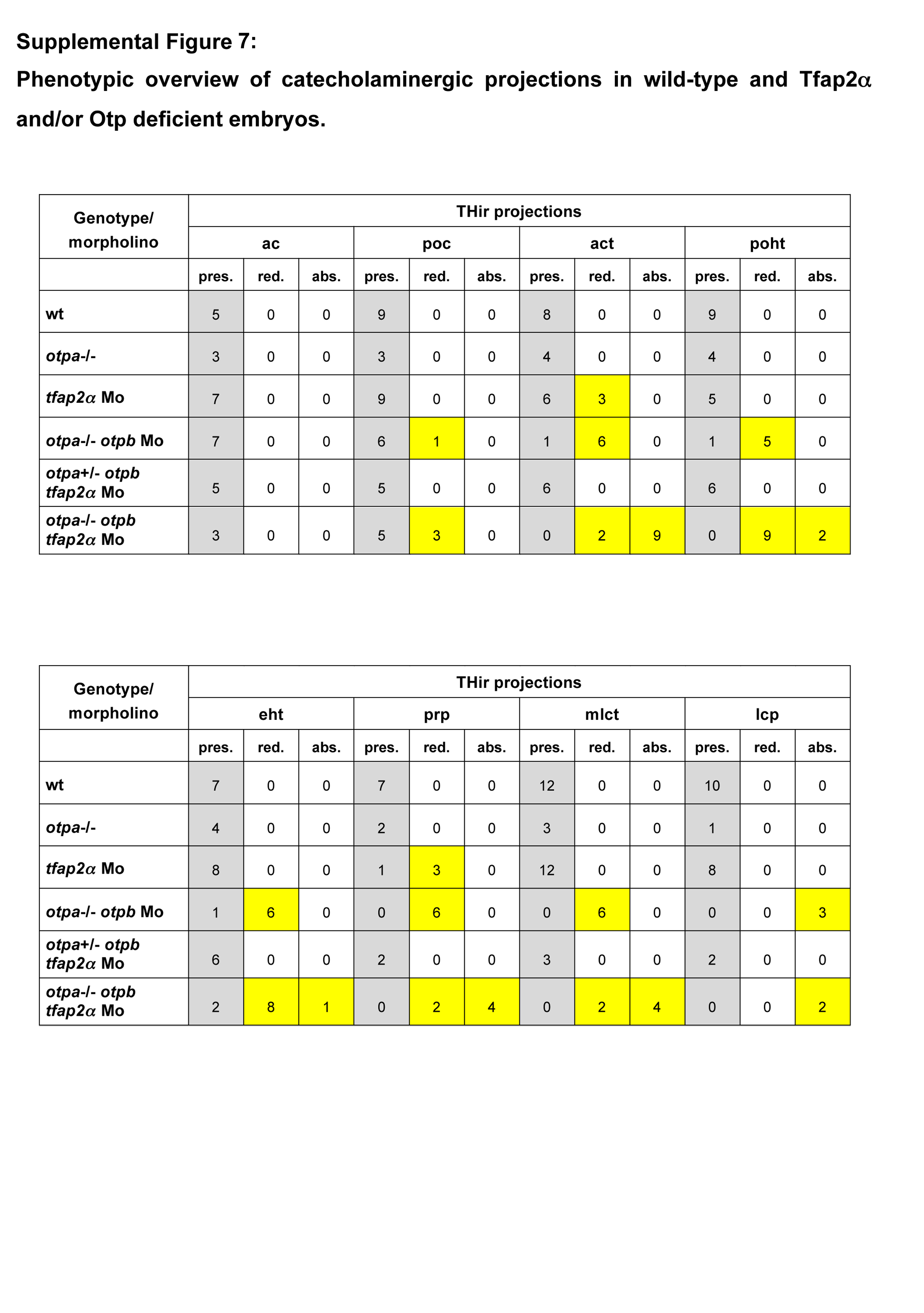

Supplement: Supplementary file 7 [file cne0518-0439-SD7.tif]
